# Tumor growth of neurofibromin-deficient cells is driven by decreased respiration and hampered by NAD^+^ and SIRT3

**DOI:** 10.1038/s41418-022-00991-4

**Published:** 2022-04-07

**Authors:** Ionica Masgras, Giuseppe Cannino, Francesco Ciscato, Carlos Sanchez-Martin, Fereshteh Babaei Darvishi, Francesca Scantamburlo, Marco Pizzi, Alessio Menga, Dolores Fregona, Alessandra Castegna, Andrea Rasola

**Affiliations:** 1 Department of Biomedical Sciences, University of Padova, via U. Bassi 58/B, 35131 Padova, Italy; 2 Institute of Neuroscience, National Research Council, via Ugo Bassi 58/B, 35131 Padova, Italy; 3 General Pathology and Cytopathology Unit, Department of Medicine-DIMED, University of Padova, via N. Giustiniani 2, 35128 Padova, Italy; 4 Department of Biosciences, Biotechnologies and Biopharmaceutics, University of Bari, via Orabona 4, 70125 Bari, Italy; 5 Department of Chemical Sciences, University of Padova, via F. Marzolo 1, 35131 Padova, Italy; 6 IBIOM-CNR, Institute of Biomembranes, Bioenergetics and Molecular Biotechnologies, National Research Council, via G. Amendola 122/O, 70126 Bari, Italy

**Keywords:** Cancer metabolism, Cell biology

## Abstract

Neurofibromin loss drives neoplastic growth and a rewiring of mitochondrial metabolism. Here we report that neurofibromin ablation dampens expression and activity of NADH dehydrogenase, the respiratory chain complex I, in an ERK-dependent fashion, decreasing both respiration and intracellular NAD^+^. Expression of the alternative NADH dehydrogenase NDI1 raises NAD^+^/NADH ratio, enhances the activity of the NAD^+^-dependent deacetylase SIRT3 and interferes with tumorigenicity in neurofibromin-deficient cells. The antineoplastic effect of NDI1 is mimicked by administration of NAD^+^ precursors or by rising expression of the NAD^+^ deacetylase SIRT3 and is synergistic with ablation of the mitochondrial chaperone TRAP1, which augments succinate dehydrogenase activity further contributing to block pro-neoplastic metabolic changes. These findings shed light on bioenergetic adaptations of tumors lacking neurofibromin, linking complex I inhibition to mitochondrial NAD^+^/NADH unbalance and SIRT3 inhibition, as well as to down-regulation of succinate dehydrogenase. This metabolic rewiring could unveil attractive therapeutic targets for neoplasms related to neurofibromin loss.

## Introduction

Metabolic changes confer tumor cells the capability to adapt to mutable environmental conditions, promoting neoplastic progression. Bioenergetic rewiring results from a complex interplay of factors intrinsic to the tumor type or enacted by interactions with heterotypic cellular and matrix components [1]. Mitochondria sense and integrate these fluctuating signals and orchestrate adaptive metabolic responses necessary to provide building blocks to the rapidly proliferating cancer cells, while shielding them from ROS damage and hypoxic conditions [2–4].

Oncogenic hyperactivation of Ras-driven transduction pathways influences mitochondrial bioenergetics by down-regulating oxidative phosphorylation (OXPHOS), eliciting anaplerotic activation of glutamine utilization as carbon source for tricarboxylic acid (TCA) cycle and inducing the (pseudo)hypoxic transcriptional program coordinated by HIF1 [1, 2, 5, 6]. Aberrant induction of Ras/MEK/ERK signaling is mandatory for neoplastic growth in Neurofibromatosis type 1 (NF1) [7], a tumor-predisposing genetic disorder caused by loss of function mutations of the NF1 gene encoding the Ras-GAP (GTPase-activating protein) neurofibromin [8]. NF1 patients are prone to develop diverse tumor types and are hallmarked by the onset of benign neurofibromas that affect Schwann cells and can evolve to extremely aggressive MPNSTs (malignant peripheral nerve sheath tumors) [7]. Transformation of neurofibromas into MPNSTs is accompanied by an increased avidity for glucose [9, 10], suggesting an enhancement of glycolysis that is paralleled by an OXPHOS down-regulation. These observations point towards a crucial role of metabolic rewiring in neoplastic progression of NF1-related tumors. Oncogenic changes in cell bioenergetics could occur by regulating the activity of key metabolic enzymes, by changing either their expression level or their activity through post-translational modifications (PTMs). We have previously demonstrated that ERK-dependent phosphorylation of the mitochondrial chaperone TRAP1 decreases the activity of the OXPHOS/TCA cycle enzyme succinate dehydrogenase (SDH) in NF1 null cells. The consequent increase in intracellular succinate levels stabilizes HIF1α and installs a pseudohypoxic transcriptional program required for NF1-related tumor growth [11, 12].

Hence, PTMs of mitochondrial proteins can tune complex pro-neoplastic bioenergetic adaptations. Further PTMs that modulate cell metabolism are provided by sirtuins (SIRTs), protein deacylases that require the metabolic cofactor nicotinamide adenine dinucleotide (NAD^+^) [13, 14]. The NAD^+^/NADH ratio shapes glycolysis, TCA cycle, OXPHOS and fatty acid oxidation; moreover, NAD^+^ is the precursor for NADP^+^ and NADPH, which display important antioxidant and biosynthetic functions [15]. Hence, NAD^+^-dependence make sirtuins cellular metabolic sensors that couple the bioenergetic status with signaling outputs affecting tumorigenicity [16].

In mitochondria, SIRT3 activates enzymes of TCA cycle, OXPHOS and fatty acid oxidation, thus increasing their functional coordination for ATP synthesis [17, 18]. SIRT3 is upregulated in some types of cancers [19], where it has been referred to as an oncogene preventing apoptosis and promoting cell proliferation [20]. Conversely, tumor growth is favored by SIRT3 ablation in several mice models, and a tumor suppressor role of SIRT3 has been reported in breast cancer, hepatocellular carcinoma, metastatic ovarian cancer and B-cell malignancies [17, 21]. This dichotomous role of SIRT3 in cancer progression could depend on the type, stage and microenvironment of the tumor, but also on NAD^+^ availability. Moreover, SIRT3 is antioxidant, as it activates both the ROS-scavenging enzyme manganese superoxide dismutase (MnSOD) and isocitrate dehydrogenase (IDH) that generates NADPH [17, 22]. The increase in ROS caused by loss of SIRT3 leads to HIF1α stabilization, making SIRT3 an antagonist of the (pseudo)hypoxic, pro-neoplastic phenotype mastered by HIF1α [23].

In this study, we report that absence of neurofibromin lowers the quantity and activity of complex I of the electron transport chain, the entry point for high-energy electrons from NADH into OXPHOS [24], thus diminishing intracellular NAD^+^/NADH ratio. Increasing intracellular NAD^+^ and reactivating SIRT3 synergize with TRAP1 inhibition in ablating neoplastic growth of NF1-related malignancies, revealing a potential therapeutic option grounded on dissection of their metabolic features.

## Materials and methods

### Nf1^−/−^ MEFs and MPNST cell lines

Mouse embryonic fibroblasts (MEFs) were derived from both wild type mice and syngenic neurofibromin 1 (Nf1)-knockout animals (Nf1^+/+^ and Nf1^−^^/−^ MEFs, respectively) [25] and were kindly provided by Dr. R. Stein, University of Tel Aviv, Ramat Aviv, Israel. sMPNST cells were established from Nf1- and p53-deficient skin precursors (SKP) [26]; cisMPNST cells were derived from spontaneous MPNSTs arising in cis Nf1^+/−^;P53^+/−^ mice [27]; both mouse MPNST cells lines were kindly provided by Dr. Lu Q. Le, University of Texas Southwestern Medical Center, Dallas, TX. Human plexiform neurofibroma ipNF 04.4 cells were generated and provided by Dr. Margaret R. Wallace, University of Florida, College of Medicine, Gainesville, FL [28]. All cells were periodically tested for mycoplasma contamination and grown in Dulbecco’s modified Eagle’s medium (DMEM) supplemented with 10% fetal bovine serum, 2 mM glutamine, 1 mM sodium pyruvate and 100 µg/ml penicillin and streptomycin at 37 °C in a humidified atmosphere containing 5% CO_2_.

### Generation of TRAP1 and SIRT3 knock-out cell lines

TRAP1 knock-out cells were generated by using the clustered regulatory interspaced short palindromic repeat (CRISPR)-Cas9 gene system [29]. Sequences for the single guides (for mouse TRAP1: 5'- CACCGCGCCGAACTCCAGCCAGCGC-3' and 5'-CACCGTTTGTGTGGGGCCCCTAAAC-3'; for mouse SIRT3: 5'-CACCGTCTATACACAGAACATCGAC-3' and 5'-CACCGTTGCTGTAGAGGCCGCTCCC-3' and 5'-CACCGACATTGGGCCTGTAGTGCCC-3') were obtained by using the CRISPR design tool (http://www.crispr.mit.edu). Scrambled single guide targeting EGFP gene was used as negative control. Oligonucleotide pairs were annealed and cloned into the transfer plasmid lentiCRISPRv2 (Addgene, Watertown, MA, USA, #52961) and co-transfected with the packaging plasmids pMDLg/pRRE (Addgene, #12251), pRSV-Rev (Addgene, #12253) and pMD2.G (Addgene, #12259) into human embryonic kidney (HEK) 293T cells for viral production. Recombinant virus was collected and used to infect cells by standard methods. Infected cells were then selected with 2 µg/ml puromycin.

### Generation of silenced and overexpressing cell lines

NF1-GRD sequence was introduced in Nf1^−/−^ MEFs using the pMSCV-GRD vector [25]. pBABE vectors were used for expression of constitutively active (CA: S217E/S221E) and dominant negative (DN: S217A) MEK1 and hyperactive RAS (G12D mutant) (Addgene, #58902). For SIRT3, SIRT4 and SIRT5 overexpression, pcDNA3.1-SIRT3/SIRT4/SIRT5 (Addgene, #13814/13815/13816) were used for sub-cloning sirtuin genes into a pBABE vector. pWPI vector was used for NDI1 expression [30] and pFUGW was used for overexpression of GFP (as a control), SIRT3 and SOD2 [31]. Murine HIF1α was silenced using the pLKO vector (Sigma) harboring shRNA against two different target sequences (#20: CCCATTCCTCATCCGTCAAAT; #22: TGGATAGCGATATGGTCAATG). HIF1α overexpression was achieved using the pBABE vector harboring either the human wild-type (Addgene, #19365) or the mutated P402A/P564A protein (Addgene, #19005). Retroviral or lentiviral vectors were used for the production of viral particles and infected cells were then selected with 2 µg/ml puromycin.

### Mitochondria isolation

Mitochondria were isolated after cell disruption with a glass-Teflon or electrical potter (Merck Sigma-Aldrich, Milano, Italy) in a buffer composed of 250 mM sucrose, 10 mM Tris-HCl, 0.1 mM EGTA-Tris, pH 7.4. Nuclei and plasma membrane fractions were separated by a first mild centrifugation (700 *g*, 10 min); mitochondria were then spinned down at 7000 *g*, 10 min, and washed twice (7000 *g*, 10 min each). All procedures were carried out at 4 °C.

### Western immunoblots and immunoprecipitations

For Western immunoblots analyses, cells or isolated mitochondria were lysed at 4 °C in a buffer composed of 150 mM NaCl, 20 mM Tris-HCl pH 7.4, 5 mM EDTA, 10% glycerol, 1% Triton X-100 (lysis buffer), in the presence of phosphatase and protease inhibitors (Merck Sigma-Aldrich). Lysates were then cleared with a centrifugation at 18,000 *g* for 30 min at 4 °C, and proteins were quantified using a BCA Protein Assay Kit (Thermo Fisher Scientific, Monza, Italy).

Protein immunoprecipitations were carried out on 200 µg isolated mitochondria. Lysates were pre-cleared with an incubation with Dynabeads^®^ Protein G (Thermo Fisher Scientific) for 1 h at 4 °C and then incubated in agitation for 18 h at 4 °C with the antibody conjugated to fresh Dynabeads^®^ Protein G. Where indicated, an unrelated anti mouse IgG was added as a negative isotype control. Beads were then washed several times in the lysis buffer.

Proteins extracted from total cell or mitochondrial lysates or from immunoprecipitations were then boiled for 5 min in Laemmli sample buffer, separated in reducing conditions on SDS-polyacrylamide gels and transferred onto Hybond-C Extra membranes (GE Healthcare Amersham, Milano, Italy) following standard methods. Primary antibodies were incubated 16 h at 4 °C, and horseradish peroxidase-conjugated secondary antibodies were added for 1 h at room temperature. Proteins were visualized by enhanced chemiluminescence (Merck Millipore, Milano, Italy).

### Blue native polyacrylamide gel electrophoresis (BN-PAGE)

BN-PAGE experiments were performed on mitochondria isolated as described above. ETC complexes and supercomplexes were extracted at 4 °C for 2 min in the presence of 2% *n*-dodecyl-β-D-maltoside (DDM) and 2% digitonin, respectively, starting from 200 μg of mitochondria in a buffer composed of 1 M aminocaproic acid, 50 mM Bis Tris pH 7. After extraction mitochondria were spinned at 100,000 *g* for 30 min and supernatants were collected and loaded on polyacrylamide Native-PAGE 3–12% Bis-Tris gradient gels (Thermo Fisher Scientific) after addition of Coomassie Blue G250 (Thermo Fisher Scientific). Protein complexes were then visualized after 18 h of Coomassie Blue G-250 staining and/or subjected to in gel activity assay (see below). Bands corresponding to the indicated respiratory chain complexes were cut and subjected to 2D-SDS-PAGE, in order to separate single protein components, which were identified by Western immunoblotting.

### Measurements of NADH dehydrogenase activity

Mitochondrial enriched fractions (20–40 µg per trace) or total protein extracts were used for spectrophotometric recordings of NADH dehydrogenase activity of mitochondrial complex I. The rotenone-sensitive NADH-CoQ oxidoreductase activity was detected following the decrease in absorbance due to the oxidation of NADH at 340 nm (ε = 6.2 mM^−1^ cm^−1^). Reaction was performed at 30 °C in 10 mM Tris-HCl pH 8 buffer containing 5 µM alamethicin, 3 mg/ml BSA, 5 µM sodium azide, 2 µM antimycin A, 65 µM coenzyme Q1, and 100 µM NADH. The NADH-ubiquinone oxidoreductase activity was measured for 3–5 min before the addition of rotenone (10 µM), after which the activity was measured for an additional 3–5 min. Measurements of complex I activity were normalized for citrate synthase (CS) activity. To measure CS activity, citrate formation was determined with a spectrophotometer as an increase in absorbance at 420 nm at 37 °C (ε = 13.6 mM^−1^ cm^−1^). Reaction buffer was composed of 100 mM Tris-HCl pH 8, 0.1% Triton X-100, 100 μM 5,5’-dithiobis-(2-nitrobenzoic acid) (DTNB), 300 μM acetyl -CoA, and 500 μM oxaloacetate. In gel complex I activity was performed by incubating Blue Native gels overnight at room temperature with a solution composed of 2 mM Tris-HCl, pH 7.4, 0.1 mg/ml NADH, and 2.5 mg/ml NTB (nitrotetrazolium blue).

### SDH succinate:coenzyme Q reductase (SQR) activity

To measure the SQR enzymatic activity of succinate dehydrogenase (SDH), cells were collected at 4 °C in a buffer composed of 25 mM potassium phosphate, pH 7.2, 5 mM magnesium chloride and protease and phosphatase inhibitors. After a cycle of freezing and thawing, cell homogenates (40 µg protein per trace) were then pre-incubated for 10 min at 30 °C in a buffer containing 25 mM potassium phosphate, pH 7.2, 5 mM magnesium chloride, 20 mM sodium succinate and 10 µM alamethicin. After the pre-incubation time, 2 µM rotenone, 5 µM antimycin A and 5 mM sodium azide were added to the medium. Reaction was started after the addition of 100 µM 2,6-dichloroindophenol (DCPIP) and 65 µM coenzyme Q1. SQR enzymatic activity was recorded following the reduction of DCPIP at 600 nM (Ɛ = 19.1 mM^−1^ cm^−1^) for 20 min at 30 °C. Each measurement of SDH activity was normalized for protein amount.

### Oxygen consumption rate (OCR) experiments

OCR was assessed in real-time with the XF24 Extracellular Flux Analyzer (Agilent, North Billerica, MA, USA). Cells (2 × 10^4^/well) were plated the day before the experiment in a DMEM/10% serum medium; experiments were carried out on confluent monolayers. Before starting measurements, cells were placed in a running DMEM medium (supplemented with 25 mM glucose, 2 mM glutamine, 1 mM sodium pyruvate, and without serum and sodium bicarbonate) and pre-incubated for 1 h at 37 °C in atmospheric CO_2_. OCR values were then normalized for the protein content of each sample. An accurate titration with the uncoupler FCCP was performed for each cell type, in order to utilize the FCCP concentration (0.5–1 µM, depending on the cell type) that maximally increases OCR.

### Measurement of the NAD^+^/NADH ratio

To measure the NAD^+^/NADH ratio in mitochondria, cells were cultured in standard conditions prior to mitochondria isolation that was performed as described above. The NAD^+^/NADH ratio was measured using the NAD^+^/NADH colorimetric assay kit (Abcam, Waltham, MA, USA), according to the manufacturer’s instructions. Briefly, isolated mitochondria were lysed by two freeze/thaw cycles in NAD^+^/NADH Extraction Buffer and vortexed for 10 seconds. After centrifugation, half supernatant was used for measurement of NADt (total amount of NAD^+^ and NADH), and the other half was used for measurement of NADH (after decomposition of NAD^+^ at 60 °C for 30 min). Samples were incubated for 5 min with NAD Cycling Mix, followed by NADH Developer solution for 4 h. Absorbance at 450 nm was then measured. The NAD^+^/NADH ratio was calculated as follows: (NADt - NADH)/NADH.

### Mass spectrometry analysis

NAD^+^/NADH ratio was calculated on tumor samples by mass spectrometry-based analysis. Tumor tissue specimens were flash frozen, weighted, and homogenized in 1 mL of 80% methanol. Samples were centrifuged at 20,000 × *g* for 10 min at 4 °C and the supernatants were transferred in a clean tube and dried using Speedvac (Thermo Fisher Scientific). Protein pellets were kept for BCA/protein assay to be used for normalization. Dried samples were reconstituted with mQ water and frozen at −80 °C. Mass spectrometry analysis was carried out with an LC-MS/MS (Quattro Premier interfaced with an Acquity UPLC system, Waters, Milford, MA, USA). The multiple reaction monitoring transition monitored for NAD^+^ was m/z 664.2 > 428.2 and for NADH m/z 666.2 > 649.1. Chromatographic resolution of NAD^+^ and NADH was achieved using an Atlantis dC18 column (2.1 150 mm, 5-m particle size, Waters) eluted with a linear gradient from 100% 10 mM ammonium formate (initial phase) to 10% 10 mM ammonium formate/90% methanol [32]. The flow was set at 0.3 ml/min. Calibration curves were established using standards, processed in the same conditions as the samples, at four concentrations [33, 34]. The lines of best fit were determined using regression analysis based on the peak area of the analytes.

### Quantitative RT-PCR

Total RNA was isolated from cells using TRIzol reagent according to the manufacturer’s instructions. 2 µg of total RNA was used to synthesize cDNA with the SuperScript III First-Strand Synthesis System (Thermo Fisher Scientific) according to the manufacturer’s protocol. Quantitative RT-PCR was performed with the Biorad qRT-PCR machine using SYBR Green (Thermo Fisher Scientific). All reactions were performed for at least 6 biological replicates and the values expressed as fold increase in mRNA levels relative to control cells. Βeta-actin was used as a housekeeping gene. qRT-PCR primers are listed in Table S1.

### Measurement of ROS

Measurements of mitochondrial ROS were performed by MitoSOX (Thermo Fisher Scientific) staining according to manufacturer’s instructions followed by flow cytometry recordings. Briefly, cells were incubated with 2.5 μM MitoSOX for 15 min at 37 °C in DMEM media depleted of FBS; next, treatments (e.g. rotenone, AUL12, antimycin A, etc.) were added and kept for the following 30–45 min. Then, cells were detached, resuspended in a buffer containing 135 mM NaCl, 10 mM HEPES and 5 mM CaCl_2_ (FACS mix solution) and analyzed. Changes in forward and side light scatter were assessed at the same time to measure alterations in cell dimension and granularity, respectively. Samples were analyzed on a FACSCanto II flow cytometer (Becton Dickinson, Franklin Lakes, NJ, USA). Data acquisition and analysis were performed using FACSDiva software.

### Cell viability assays

Cell viability was assessed either by a colorimetric MTS assay (Cell Titer 96^®^ Aqueous One Solution; Promega, Madison, WI, USA) or by flow cytometry analysis. For MTS measurements, plates were incubated at 37 °C overnight and read in a microplate spectrophotometer (Infinite^®^ 200 PRO, Tecan Life Sciences, Mannedorf, Switzerland). Flow cytometry recordings were performed as described previously [35, 36]. Briefly, cells were stained with FITC-conjugated Annexin-V and 7-Aminoactinomycin D (7-AAD) to determine phosphatidylserine exposure on the cell surface (increased FITC-conjugated Annexin-V staining) and loss of plasma membrane integrity (7-AAD permeability and staining). Cells were incubated at 37 °C in an assay buffer containing 135 mM sodium chloride, 10 mM HEPES, 5 mM calcium chloride and samples were then analyzed on a FACS Canto II flow cytometer (Becton Dickinson). Data acquisition and analysis were performed using FACSDiva software.

### In vitro tumorigenesis assays

Focus forming assays were performed on cells grown in 12-well culture plates in DMEM medium supplemented with 10% fetal bovine serum. When cells reached sub-confluence, serum concentration was reduced to 1% and NIC was added at a concentration of 5 mM. At the 3rd or 5th day after serum decrease, cells were scraped and collected at 4 °C and SQR enzymatic activity of SDH was measured as described above. For the soft agar assay, cells were grown in 24 well plates covered by a bottom layer composed of DMEM medium mixed with low melting point agarose (Promega) at a final concentration of 1%, and by a top layer of DMEM medium supplemented with 1% serum and mixed with low melting point agarose at a final concentration of 0.6%. Cells (0.2 × 10^5^/cm^2^) were added during the preparation of the upper layer, where they remained embedded. Dishes were then maintained in a humidified atmosphere of 5% CO_2_-95% air at 37 °C for 3 weeks, adding medium (DMEM with 2% serum) on the top of the two layers every 3rd day. At the 25–30th day, dishes were washed in PBS and colonies were stained with Crystal Violet 0.005% and analyzed with ImageJ software. Growth in 4% Matrigel (Corning, New York, NY, USA) was performed in low adhesion 24 well plates in DMEM medium supplemented with 2% FBS. Cells (0.1 × 10^5^/cm^2^) were seeded in 600 μl final volume and after 2–3 days colonies were stained with Crystal Violet 0.005% and analyzed with ImageJ software.

### In vivo tumorigenesis assays

Experiments were performed in 8-week-old nude female mice (Charles River, Wilmington, MA). All mice were housed on a 12:12 h light:dark cycle at 25 °C in accordance with the European Community guidelines. Eight-week-old animals (*n* ≥ 7) were injected subcutaneously bilaterally in the flanks with 1.5 × 10^6^ sMPNST in 100 μl of serum-free sterile PBS mixed with 4% Matrigel. Nicotinic acid treatment (1% into drinking water) was administered at day 2 following xenograft injection and refreshed every 3 days. Tumors were visible under the skin after 7–9 days and measured with a caliper every 4 days (two major axes). Tumor volume was calculated using the formula: (length × width^2^)/2. After 3 weeks, mice were sacrificed and tumors stored at −80 °C or fixed in formaldehyde and maintained in 70% ethanol for immunohistochemical analyses.

### Immunohistochemical analyses

Histological and immunohistochemical analyses were performed on samples derived from mouse tumor grafts and all analyzed parameters were blindly evaluated by the same pathologist. In detail, 4 µm-thick tissue sections were obtained from formalin-fixed paraffin-embedded tissue samples and representative tumor areas were selected on H&E-stained slides for immunohistochemical (IHC) analysis. IHC was performed using a primary rabbit polyclonal anti HIF1α antibody (Novus Biologicals, Centennials, CO, USA). Antigen retrieval was performed with heat/EDTA in the Bond-Max automated immunostainer (Leica Biosystems, Wetzlar, Germany), as previously described [37].

### Quantification and statistical analysis

Data were analyzed and presented as mean ± standard deviation (SD) or standard error of the mean (SEM) in all figures. Pairs of data groups were analyzed using paired and unpaired two-tailed Student’s *t* tests. In the case of more than two groups, one-way analysis of variance (ANOVA) followed by Bonferroni post-hoc test was applied. Statistical significance was determined using Origin^®^ 8 (OriginLab, Northampton, MA). Results with a *p* value lower than 0.05 were considered significant; ***p < 0.001, **p < 0.01, *p < 0.05 compared to controls. Each experiment was repeated at least three times.

## Results

### Neurofibromin loss decreases protein levels and enzymatic activity of NADH dehydrogenase in an ERK1/2 dependent manner

**Fig. 1:**
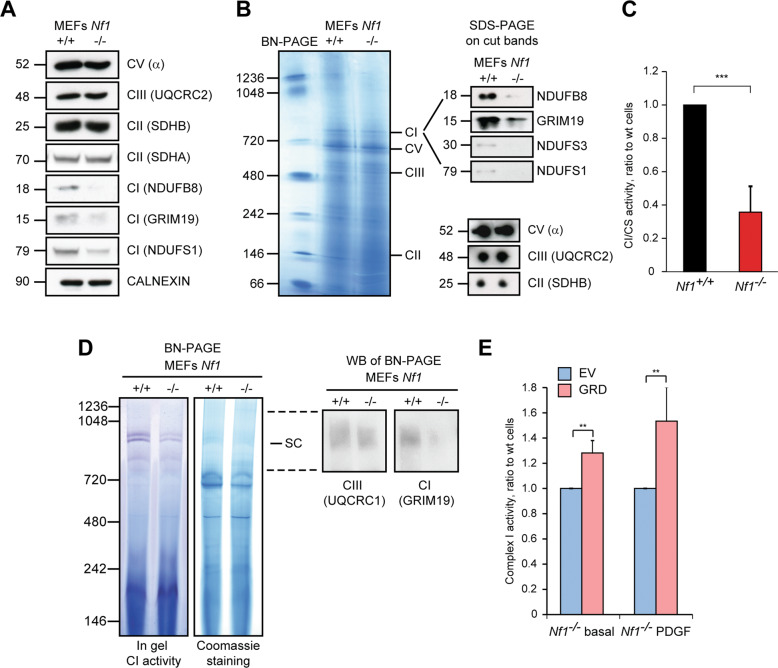
The absence of neurofibromin decreases protein levels and enzymatic activity of respiratory complex I. OXPHOS protein levels were analyzed by Western immunoblot (WB, **A**) or blue native polyacrylamide gel electrophoresis (BN-PAGE, **B**). In **A**, calnexin was used as a loading control. In **B**, bands corresponding to different respiratory complexes were cut, run on an SDS-PAGE and probed for the expression of the complex I subunits NDUFB8, GRIM19, NDUFS1 and NDUFS3, of the complex V α subunit, of the complex III UQCRC2 subunit and of the complex II SDHB subunit. **C** Spectrophotometric analysis of the NADH dehydrogenase activity of complex I (CI) is shown as arbitrary units and normalized for citrate synthase (CS) activity. **D** BN-PAGE carried out on digitonized mitochondria to preserve assembled respiratory supercomplexes. Gels were either subjected to in gel complex I activity, or stained with Coomassie blue and transferred to PVDF membrane for protein identification. UQCRC1 and GRIM19 are complex III and complex I subunits, respectively. **E** Spectrophotometric analysis of the NADH dehydrogenase activity of complex I in control (empty vector, EV) and GRD Nf1^−/−^ MEFs in basal condition (10 % FBS) or following growth in a media containing PDGF (10 ng/ml, and 0.5 % FBS). All experiments in the Figure were carried out in Nf1^+/+^ and Nf1^−/−^ MEFs. Data are reported as mean ± SD values (*n* ≥ 3); ***p < 0.001; **p < 0.01; *p < 0.05 with a Student’s *t* test analysis.

We have explored the possibility that neurofibromin loss could affect cell bioenergetics by regulating expression and/or activity of mitochondrial OXPHOS complexes. By comparing mouse embryonic fibroblasts (MEFs) derived from wild type and Nf1^−/−^ animals [25], we have found that Nf1^−/−^ MEFs have a lower expression and activity of OXPHOS complex I (*aka* NADH dehydrogenase or NADH-ubiquinone oxidoreductase) than their wild type counterparts (Fig. 1A–C). Similarly, Nf1^−/−^ MEFs exhibit a decrease of complex I expression and activity in respiratory supercomplexes (SCs) (Fig. 1D) that functionally link OXPHOS complexes to funnel electron transfer along the respiratory chain [38, 39]. Expression of the GAP-related domain of neurofibromin (NF1-GRD), which reverses Ras activation in Nf1^−/−^ MEFs [25], rescues complex I activity (Fig. 1E) while the constitutively active Ras^G12D^ mutant decreases it in wild type MEFs, mimicking the effect of neurofibromin ablation (Supplementary Fig. 1A).

**Fig. 2:**
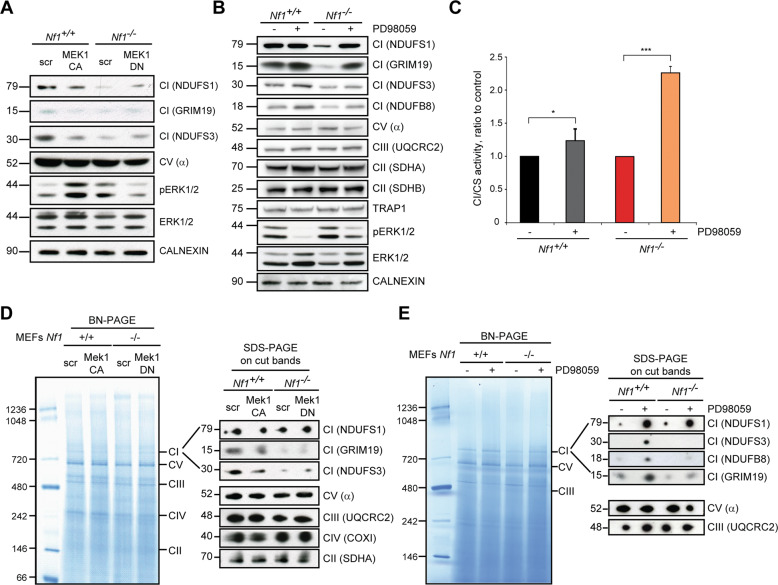
Respiratory complex I expression and activity are regulated in a MEK/ERK-dependent fashion. WB analyses of Complex I subunits upon modulation of ERK activity through expression of the constitutively-active or dominant-negative form of the upstream MEK1 kinase (MEK1-CA and MEK1-DN, respectively; **A**) or by treatment with the MEK inhibitor PD98059 (40 µM, 3 days; **B**). NDUFS1, GRIM19, NDUFS3 and NDUFB8 were used as complex I markers, subunits α, UQCRC2 and SDHA/B as complex V, complex III and complex II markers, respectively. pERK1/2 indicates phosphorylated, active ERK1/2. TRAP1 and calnexin were used as loading controls. **C** Complex I activity with or without PD98059 treatment (40 µM, 3 days) was analyzed as in Fig. 1C. **D**, **E** BN-PAGE of respiratory complexes performed on digitonized mitochondria from Nf1^+/+^ and Nf1^−/−^ MEFs. The MEK-ERK pathway is modulated by expression of the constitutively-active or of the dominant-negative form of the upstream MEK1 kinase (MEK1-CA and MEK1-DN, respectively; **D**) or by treatment with the MEK inhibitor PD98059 (40 µM, 3 days; **E**). All experiments in the Figure were carried out on Nf1^+/+^ and Nf1^−/−^ MEFs. Data are reported as mean ± SD values (*n* ≥ 3); ***p < 0.001; **p < 0.01 and *p < 0.05 with a Student’s *t* test analysis.

This down-regulation of complex I is elicited by Ras/MEK/ERK signaling activation, as demonstrated by expression of a constitutively active MEK1 (MEK1-CA) kinase, whereas ERK inhibition by a dominant-negative MEK1 (MEK1-DN) protein, as well as by the MEK inhibitor PD98059, enhances both protein levels and enzymatic function of NADH dehydrogenase (Fig. 2A–E; Supplementary Fig. 1B). MEK inhibition correlates with an increase in complex I expression also in human U87 glioblastoma cells (Supplementary Fig. 1C) and ipNF 04.4 plexiform neurofibroma cells (Supplementary Fig. 1D), both characterized by absence of neurofibromin and hence by Ras/MEK/ERK pathway induction. Protein levels of the other OXPHOS complexes are unaffected by hyperactivation of Ras/MEK/ERK signaling in all these cell models (Figs. 1A, B and 2B, D, E; Supplementary Fig. 1C, D). Neurofibromin loss does not elicit differences in mRNA expression of complex I subunits (Supplementary Fig. 1E). These results connect deregulated activation of Ras/MEK/ERK signaling caused by absence of neurofibromin with complex I inhibition, in keeping with a reported role of oncogenic Ras in orchestrating the metabolic rewiring of tumor cells [6].

### The alternative NADH dehydrogenase NDI1 increases oxygen consumption rate and confers resistance to oxidative stress in neurofibromin-expressing cells

**Fig. 3:**
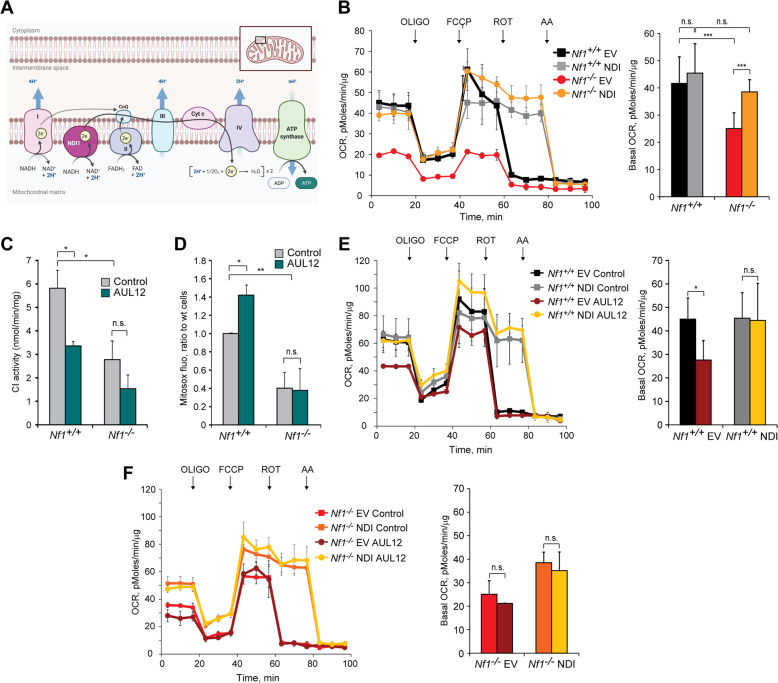
Alternative NADH dehydrogenase NDI1 rescues oxygen consumption rate (OCR) inhibition and protects from toxicity of complex I inhibitors. **A** Scheme of NDI1 function when expressed in mammalian cells characterized by defective or inactivated NADH dehydrogenase. **B**, **E**, **F** Representative OCR traces and quantification of basal OCR of Nf1^+/+^ and Nf1^−/−^ MEFs. The ATP synthase inhibitor oligomycin (0.8 µM), the proton uncoupler carbonyl cyanide-4-(trifluoromethoxy)phenylhydrazone (FCCP, 1 µM) and the respiratory complex I and III inhibitors rotenone (0.5 µM) and antimycin A (1 µM), respectively, were added as indicated. In **E**, **F**, AUL12 (4 µM) was added 45 min before recordings. NDI: cells expressing the pWPI-NDI1 construct; EV: cells expressing the pWPI empty vector. **C** Measurement of complex I activity in control and AUL12-treated mitochondria. **D** Analysis of mitochondrial ROS levels by MitoSOX staining in control and AUL12-treated (4 mM, 1 h) cells. All experiments in the Figure were carried out on Nf1^+/+^ and Nf1^−/−^ MEFs. Data are reported as mean ± SD values (*n* ≥ 3); ***p < 0.001; **p < 0.01 and *p < 0.05 with a Student’s *t* test analysis or One-way ANOVA followed by Bonferroni post-test (**B**–**D**).

We have then investigated possible mechanistic links between complex I inhibition and downstream effects on tumorigenicity of Nf1^−/−^ cells. To this aim, we have bypassed complex I function by expressing NADH dehydrogenase 1 (NDI1; Supplementary Fig. 2A), a single subunit enzyme from *S. cerevisiae* that catalyzes electron transfer from NADH to ubiquinone, without proton translocation and in a rotenone-insensitive way [30, 40] (Fig. 3A). OCR is down-regulated in cells lacking neurofibromin (Fig. 3B). GRD expression rescues this inhibition (Supplementary Fig. 2B), indicating that it occurs downstream to Ras hyperactivation. When expressing NDI1, Nf1^−/−^ MEFs increase their OCR and reach the level of Nf1^+/+^ cells, which instead are not affected by NDI1 (Fig. 3B). Expectedly, NDI1 makes MEFs refractory to changes in OCR caused by the selective complex I inhibitor rotenone (Supplementary Fig. 2C).

Nf1^+/+^ MEF cells display a higher level of mitochondrial ROS than Nf1^−/−^ MEFs (Supplementary Fig. 3A), in accord with their higher activity of complex I (Fig. 1C, D), one of the major sources of cellular ROS [41]. Rotenone exacerbates the difference in mitochondrial ROS levels between Nf1^−/−^ and Nf1^+/+^ MEF cells (Supplementary Fig. 3A) and elicits oxidative stress toxicity only in the latter ones (Supplementary Fig. 3B). NDI1 blunts the increase in mitochondrial ROS induced by rotenone (Supplementary Fig. 3C) and the consequent death of Nf1^+/+^ MEFs (Supplementary Fig. 3D). Another complex I inhibitor, gold-dithiocarbamate complex AUL12 (dibromo [ethyl-*N*-(dithiocarboxy-kS,kS′)-*N*-methylglycinate] gold(III)), can induce a noxious mitochondrial ROS surge in tumor cells [42, 43]. Exposure to AUL12 inhibits NADH dehydrogenase and increases mitochondrial ROS, causing oxidative-stress dependent death, only in Nf1^+/+^ MEF cells (Fig. 3C, D; Supplementary Fig. 3E), consistently with their high complex I activity. AUL12 lowers OCR only in neurofibromin-expressing cells, making them reach the same level of Nf1^−/−^ MEFs (Fig. 3E, F). NDI1 circumvents this OCR inhibition (Fig. 3E, F) and protects Nf1^+/+^ MEFs from AUL12-induced ROS surge and cell death (Supplementary Fig. 3F, G).

Together, these observations indicate that complex I inhibition is responsible for the lower respiratory rate of cells lacking neurofibromin with respect to Nf1^+/+^ cells. NDI1 blunts the increase in mitochondrial ROS and the consequent toxicity of complex I targeting compounds in Nf1^+/+^ MEFs, and constitutes a tool to investigate the impact of complex I inhibition on the tumorigenic potential of cells lacking neurofibromin.

### NDI1 impairs tumorigenicity of neurofibromin-deficient cells through SIRT3 reactivation

**Fig. 4:**
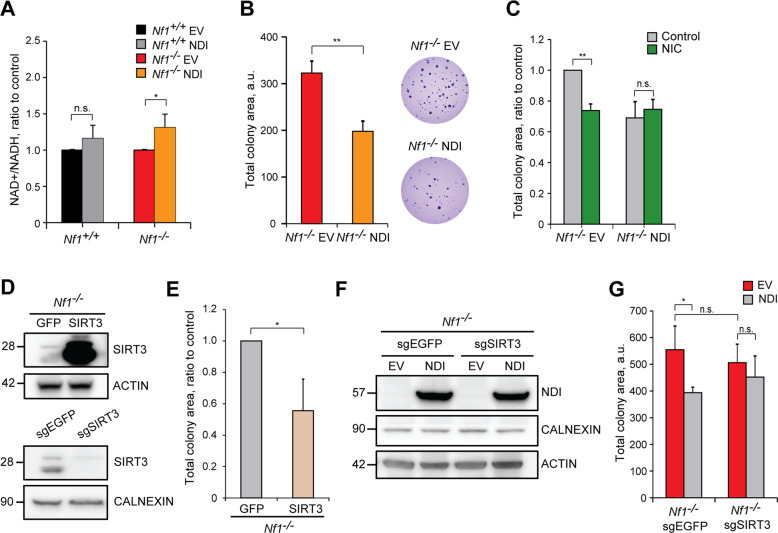
The NDI1-NAD^+^-SIRT3 axis impairs tumorigenicity of neurofibromin-deficient cells. **A** Spectrophotometric analysis of the NAD^+^/NADH ratio following NDI1 expression in Nf1^+/+^ and Nf1^−/−^ MEFs. **B** Effect of NDI expression on soft agar growth of Nf1^−/−^ cells. Measurement of colony area (left) and representative pictures of colonies (right) are reported. NDI: cells expressing the pWPI-NDI1 construct; EV: cells expressing the pWPI empty vector. **C** Effect of nicotinic acid (NIC 5 mM, 3 weeks) on colony growth in soft agar of Nf1^−/−^ cells. **D** WB analysis of SIRT3 protein levels after expression of the pFUGW-GFP/SIRT3 construct (upper part) or SIRT3 knocking-out (sgSIRT3, lower part). Negative controls were cells expressing pFUGW-GFP (GFP, upper part) or scrambled single guide targeting EGFP (sgEGFP, lower part). **E** Effect of SIRT3 upregulation on soft agar colony formation of Nf1^−/−^ cells. **F** WB analysis of NDI1 expression in SIRT3 wild-type and knock-out (sgEGFP and sgSIRT3, respectively) neurofibromin-deficient cells. **G** Effect of NDI1 expression on soft agar growth of SIRT3 wild-type (sgEGFP) or knock-out (sgSIRT3) Nf1^−/−^ MEFs. In **D**, **F**, actin and calnexin were used as loading controls. Data are reported as mean ± SD values (*n* ≥ 3); ***p < 0.001; **p < 0.01 and *p < 0.05 with a Student’s *t* test analysis or One-way ANOVA followed by Bonferroni post-test (**G**).

Complex I activity is a master regulator of NAD^+^ and NADH intracellular levels. In Nf1^−/−^ cells, but not in the wild type ones, NDI1 expression raises intracellular NAD^+^/NADH ratio and NAD^+^ levels (Fig. 4A; Supplementary Fig. 4A), in accord with its NADH dehydrogenase activity. Although proliferation rate is not changed (Supplementary Fig. 4B), tumorigenicity of neurofibromin-deficient cells is decreased by NDI1 in a soft agar assay (Fig. 4B), which measures the capability of cells to form colonies in an anchorage-independent way. These observations highlight the possibility of a direct connection between increased NAD^+^/NADH balance and neoplastic growth driven by neurofibromin deficiency. Accordingly, the NAD^+^ precursors nicotinic acid (NIC) and nicotinamide (NAM), which augment NAD^+^ levels through the Preiss-Handler and the salvage pathway, respectively [15], decrease tumorigenicity of Nf1^−/−^ cells to levels comparable to their NDI1-expressing counterparts, on which NIC and NAM are ineffective (Fig. 4C; Supplementary Fig. 4C).

**Fig. 5:**
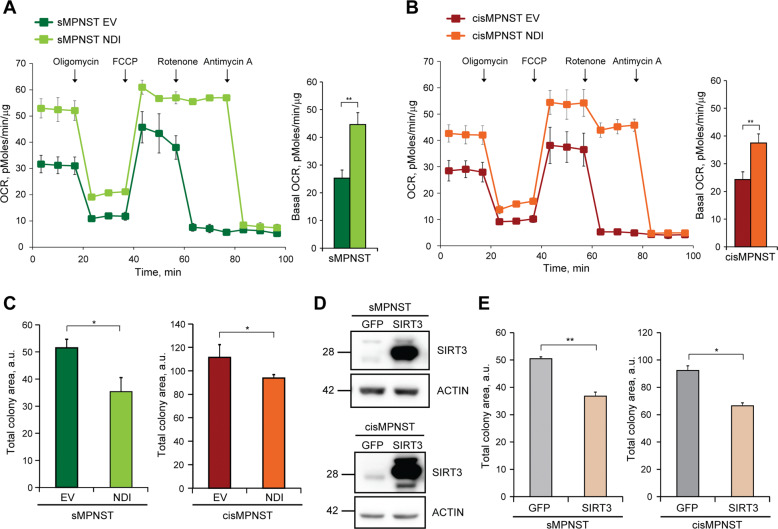
Expression of either NDI1 or SIRT3 impairs tumorigenicity of MPNST cells. Representative OCR traces and quantification of basal OCR of sMPNST (**A**) and cisMPNST (**B**) cells. The ATP synthase inhibitor oligomycin (0.8 µM), the proton uncoupler carbonyl cyanide-4-(trifluoromethoxy)phenylhydrazone (FCCP, 1 µM) and the respiratory complex I and III inhibitors rotenone (0.5 µM) and antimycin A (1 µM), respectively, were added where indicated. **C** Effect of NDI1 expression on soft agar colony formation of sMPNST (left) and cisMPNST (right) cells. SIRT3 overexpression after transfection with pFUGW-SIRT3 (**D**) decreases Matrigel colony formation of MPNST cells (**E**). In **D**, actin was used as a loading control. All along the Figure, the MPNST cell models sMPNST and cisMPNST were used. NDI: cells expressing the pWPI-NDI1 construct; EV: cells expressing the pWPI empty vector; SIRT3: cells expressing pFUGW-SIRT3; GFP: cells expressing pFUGW-GFP. Data are reported as mean ± SD values (*n* ≥ 3); ***p < 0.001; **p < 0.01 and *p < 0.05 with a Student’s *t* test analysis.

Increased mitochondrial NAD^+^ levels can enhance the activity of the mitochondrial sirtuins SIRT3, SIRT4 and SIRT5. SIRT3 overexpression in cells without neurofibromin (Fig. 4D) decreases their tumorigenicity (Fig. 4E), while SIRT4 or SIRT5 overexpression could not affect it (Supplementary Fig. 4D). Knocking-out SIRT3 (Fig. 4D) makes Nf1^−/−^ cells insensitive to the antineoplastic effect of NDI1 (Fig. 4F, G), in line with an inhibitory effect on tumor growth of NDI1 that requires SIRT3 activity. NDI1 expression does not affect the expression level of SIRT3 and of its targets SDHA, IDH2 and SOD2 (Supplementary Fig. 4E, F), but decreases SOD2 acetylation (Supplementary Fig. 4E). Deacetylation of SOD2 elicits its key antioxidant functions and is considered a bona fide indication of SIRT3 activity [22, 44]. Indeed, SIRT3 overexpression reduces SOD2 acetylation (Supplementary Fig. 4G), as well as administration of NIC or NAM (Supplementary Fig. 4H), and forcing SOD2 activity through its overexpression decreases the tumorigenic potential of neurofibromin-deficient cells (Supplementary Fig. 4I). These data are in accord with the hypothesis of a pathway connecting increased intracellular NAD^+^/NADH ratio with antineoplastic SIRT3 induction. To further corroborate this model, we have analyzed cell types derived from MPNSTs, malignancies typically associated to NF1 that are endowed with a profound metabolic rewiring [45] and for which effective therapies are lacking [46]. In accord with our observations in MEFs lacking neurofibromin, NDI1 expression increases OCR (Fig. 5A, B) and reduces tumorigenicity (Fig. 5C) in MPNST cells too, and a similar antineoplastic effect is obtained by SIRT3 overexpression (Fig. 5D, E).

These results indicate that, upon neurofibromin loss, hyperactivation of Ras/MEK/ERK signaling inhibits complex I, causing a decrease in the NAD^+^/NADH ratio and the ensuing SIRT3 repression. Raising the NAD^+^/NADH ratio *via* NDI1 expression has an antineoplastic effect through the enhancement of SIRT3 activity.

### TRAP1 ablation and NAD^+^/SIRT3 axis counteract pro-neoplastic bioenergetic adaptations of MPNST cells

We have previously shown that the mitochondrial chaperone TRAP1 has a pro-neoplastic effect by inhibiting SDH activity [12] and that this applies to NF1-related models, where TRAP1 activity is increased in an ERK-dependent way [11]. SDH constitutes a potential point of intersection between the bioenergetic effects caused by SIRT3 induction and TRAP1 ablation, as SIRT3 increases SDH activity [47, 48] similarly to TRAP1 absence or inhibition [11, 49]. However, it was also proposed that TRAP1 is activated by SIRT3-dependent deacetylation in a glioblastoma model [50], making it difficult to draw a comprehensive picture.

**Fig. 6:**
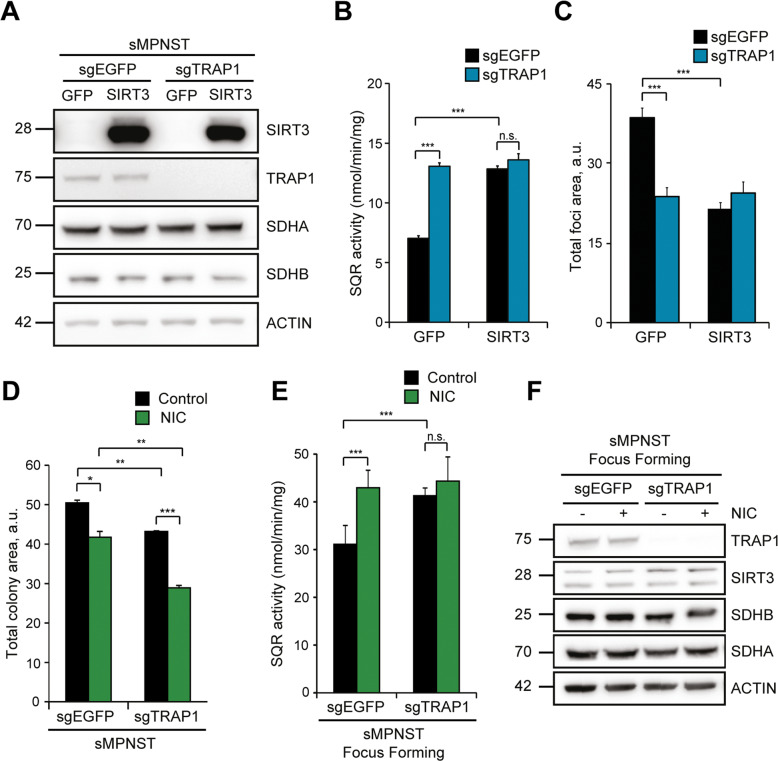
Enhancement of SDH activity by SIRT3/NIC or TRAP1 parallels inhibition of tumorigenic growth. **A** WB analysis of SIRT3 and SDHA/B protein levels in TRAP1 wild-type (sgEGFP) and knock-out (sgTRAP1) sMPNST cells. Actin was used as a loading control. **B** Analysis of succinate-coenzyme Q reductase (SQR) activity of SDH in TRAP1 wild-type and knock-out sMPNST cells upon SIRT3 overexpression. SIRT3: cells expressing pFUGW-SIRT3; GFP: cells expressing pFUGW-GFP. **C** Foci formation of sMPNST cells upon TRAP1 knock-out and SIRT3 overexpression. Effect of NIC treatment (5 mM) on Matrigel colony formation (**D**), SQR activity (**E**) and SDHA/B protein levels (**F**) of TRAP1 wild-type and knock-out sMPNST cells. Measurements in **E**, **F** were carried out on cells undergoing tumorigenic growth (day 5th of focus forming assay). In **F** actin was used as a loading control. Data are reported as mean ± SD values (*n* ≥ 3); ***p < 0.001; **p < 0.01 and *p < 0.05 with One-way ANOVA followed by Bonferroni post-test.

We have therefore studied whether there is an interplay between the pro-tumoral roles exerted by TRAP1 and by SIRT3 inhibition downstream to respiratory complex I down-regulation in NF1-related malignant cells. In both sMPNST and cisMPNST cells, neither TRAP1 knock-out nor SIRT3 overexpression change protein levels of SDH subunits (Fig. 6A; Supplementary Fig. 5A), but both conditions raise the succinate:coenzyme Q reductase (SQR) activity of SDH to a similar extent and without any additive effect (Fig. 6B; Supplementary Fig. 5B), which is strongly suggestive of a common effector mechanism. As expected [49], knocking-out TRAP1 expression reduces tumorigenicity of MPNST cells, and this is mimicked by SIRT3 overexpression (Fig. 6C; Supplementary Fig. 5C). A similar antineoplastic effect is obtained upon NIC administration to TRAP1-expressing cells, and NIC further inhibits colony growth in TRAP1 knock-out cells (Fig. 6D). In the same conditions of in vitro tumorigenicity, induction of SQR activity either by ablating TRAP1 or by supplementing NIC (Fig. 6E) matches with inhibition of colony growth (Fig. 6D) without any change in SDH or SIRT3 protein levels (Fig. 6F).

**Fig. 7:**
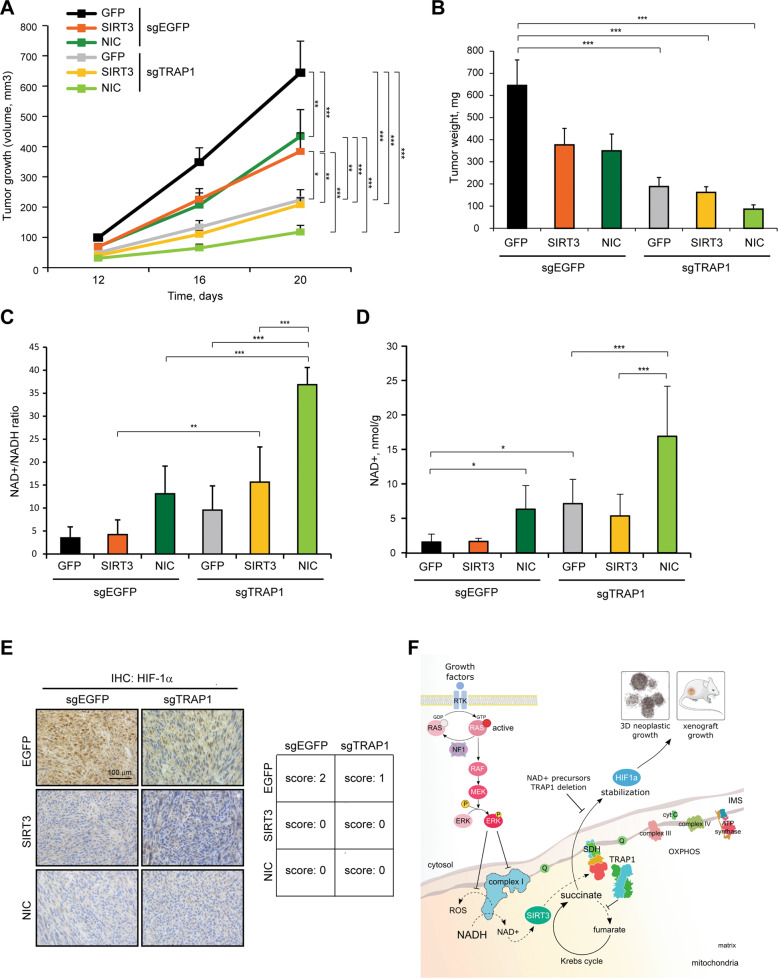
SIRT3/NIC and TRAP1 ablation hamper in vivo tumor growth. **A** Kinetics of tumor growth of wild type (sgEGFP) and TRAP1 knock-out (sgTRAP1) sMPNST cells xenografted in nude mice. SIRT3: cells expressing pFUGW-SIRT3; GFP: cells expressing pFUGW-GFP. NIC: animals treated with nicotinic acid (1% in drinking water) for the whole duration of the experiment. Tumor volume is expressed in mm^3^. Measurement of tumor weight (mg; **B**), NAD^+^/NADH ratio (**C**) and NAD^+^ (nmol/g; **D**) of tumor samples after xenograft injection in nude mice of wild type and TRAP1 knock-out sMPNST cells. In **C**, **D** data are reported as mean ± SD values (*n* ≥ 3). In **A**, **B** data are reported as mean ± SEM values (*n* ≥ 7). A two-way ANOVA followed by Bonferroni post-test was performed in **A**. One-way ANOVA followed by Bonferroni post-test were performed in **B**–**D**. ***p < 0.001; **p < 0.01 and *p < 0.05. **E** Immunohistochemical analysis of HIF1α expression in tumor samples from **A**. 40X magnification. Intensity of the signal was scored as it follows: score 0 = negative staining or weak/barely perceptible expression in <25% of cells; score 1 = weak/barely perceptible positivity in >25% of cells; score 2 = moderate to strong positivity in >25% of cells. **F** Model of the metabolic changes occurring in mitochondria following hyperactivation of the RAS/MEK/ERK pathway caused by neurofibromin ablation.

In vivo, neoplastic growth of MPNST cells is inhibited by SIRT3 overexpression or treatment of mice with NIC, as well as by TRAP1 knock-out (Fig. 7A, B). SIRT3 overexpression does not further decrease tumor growth in a TRAP1-null background, whereas NIC treatment almost abrogates it (Fig. 7A, B). In tumor samples both NAD^+^/NADH ratio and NAD^+^ are increased following NIC administration and when TRAP1 is ablated, and are dramatically raised when NIC is provided to animals harboring TRAP1 knock-out cells (Fig. 7C, D). Consistently, the activity of NADH dehydrogenase, but not its expression, is augmented in conditions of tumorigenic growth when TRAP1 is absent (Supplementary Fig. 5D, E).

We observe a marked nuclear localization of the transcription factor HIF1α in xenografted sMPNST cells (Fig. 7E), in accord with the crucial pro-neoplastic role played by a feed-forward activation loop between HIF1α and TRAP1 [12, 51], and indeed HIF1α silencing impairs tumorigenicity of sMPNST cells (Supplementary Fig. 5F). Not only TRAP1 knock-out, but also SIRT3 induction and NIC supplementation abrogate the nuclear localization of HIF1α (Fig. 7E). Moreover, overexpression of a HIF1α mutant (P402A/P577A) that does not undergo prolyl hydroxylation-dependent degradation rescues the inhibition of neoplastic growth elicited by SIRT3 overexpression (Supplementary Fig. 5G), in line with a SIRT3-mediated stabilization of HIF1α.

These findings indicate that tumorigenic growth of NF1-related neoplasms can be impaired both by TRAP1 ablation and by SIRT3 re-activation. Combination of these two approaches reaches maximal efficacy when SIRT3 induction is achieved through a rise in NAD^+^ precursors.

## Discussion

In the present study, we demonstrate that deficiency of the RasGAP neurofibromin down-modulates respiration through inhibition of NADH dehydrogenase, the respiratory complex I. We connect complex I inhibition to the specific hyperactivation of MEK-ERK signaling downstream to neurofibromin ablation and the ensuing aberrant induction of Ras. Deregulation of this pathway is mandatory for growth of NF1-related tumors [7], and our observations indicate that complex I inhibition may have a dual effect that can be exploited by the neurofibromin-deficient tumor cell. On the one hand, it keeps intracellular ROS levels at bay, shielding cells from a variety of environmental insults that can hamper neoplastic growth by eliciting oxidative stress. These may include pro-oxidant chemotherapeutics, but also hypoxic conditions encountered by cells during the turbulent growth of the tumor mass [52]. On the other hand, a low NADH dehydrogenase activity means dropping the NAD^+^/NADH ratio, and NAD^+^ acts as a co-substrate for sirtuin deacylases [13]. We find that increasing the NAD^+^/NADH balance or overexpressing the NAD^+^-dependent mitochondrial deacetylase SIRT3, but not SIRT4 or SIRT5, hinder tumorigenicity in neurofibromin-deficient cells, including highly aggressive MPNST models. Conversely, knocking-out SIRT3 does not further increase MPNST tumorigenicity, indicating that it is constitutively inhibited in neurofibromin-lacking cells. These observations point toward a bona fide tumor suppressor role for SIRT3, which is abrogated by respiratory complex I inhibition and the ensuing decrease of NAD^+^/NADH ratio in NF1-related tumor cells, and shed light on the molecular mechanisms and biochemical effects of the previously observed complex I inhibition in K-Ras transformed cells [53, 54].

The use of the yeast NADH dehydrogenase NDI1, a rotenone insensitive, single subunit protein capable of restoring NADH oxidation and mitochondrial respiration in cells devoid of complex I activity [30], increases respiration of Nf1^−/−^ cells to the levels of their neurofibromin-expressing counterparts. NDI1 impairs tumorigenicity of cells carrying a complex I dysfunction [55] and in a breast cancer model [56]. Accordingly, we observe that NDI1 thwarts tumorigenicity of NF1-related tumor cells, directly demonstrating a causal connection between a decrement in the activity of respiratory complex I and neoplastic growth. Thus, as already applied for mitochondrial diseases [57], we propose NDI1 as a useful tool to uncover NADH dehydrogenase inhibition in specific tumor cells and bypass it in order to assess its potential contribution to neoplastic transformation. In our system, NDI1 was ineffective on the tumorigenic potential of cells where we knocked-out SIRT3 expression, further underlining that NDI1 hampers neoplastic growth in a SIRT3-dependent manner.

SIRT3 works as a master regulator of mitochondrial metabolism and redox homeostasis by affecting the activity of enzymes such as glutamate dehydrogenase, isocitrate dehydrogenase 2, serine hydroxymethyltransferase 2, superoxide dismutase and pyruvate dehydrogenase [13, 58]. Moreover, it has been proposed that SIRT3 activates the mitochondrial chaperone TRAP1, thus contributing to the maintenance of cancer stem cells in a glioblastoma model [50]. We have formerly demonstrated that TRAP1 is pro-neoplastic in diverse neoplastic models by inhibiting SDH activity, thus activating HIF1α [12, 59–62], and that a mitochondrial fraction of ERK [63] phosphorylates TRAP1 and enhances its tumorigenic activity in NF1 models [11]. Consequently, it is difficult to reconcile an oncogenic function of TRAP1 with its activation by SIRT3, if the latter plays a tumor suppressor role. In this regard, we have recently found that SIRT3 overexpression raises SDH activity, mimicking the absence of TRAP1 [49]. In this study, we report that knocking-out TRAP1 expression is ineffective in further increasing SDH activity of SIRT3-overexpressing cells, in accord with SIRT3 enhancing SDH activity via TRAP1 inhibition. Therefore, we propose that SIRT3 activation in MPNST cells is antineoplastic at least in part by re-establishing SDH activity and counteracting HIF1α stabilization, as we have observed in xenografted cancer cells. Given that both SIRT3 overexpression and its induction through agents increasing NAD^+^ (NIC/NAM administration) activate SOD2, and that SOD2-overexpressing cells decrease their tumorigenicity, it is possible that SIRT3-mediated activation of SOD2 further contributes in the repression of neoplastic growth of Nf1^−/−^ cells.

We envision that enhancement of SIRT3 activity and replenishment of NAD^+^ levels result in a multifaceted metabolic rewiring, which can oppose NF1-related cancer growth by affecting multiple bioenergetic pathways. Accordingly, the antineoplastic effect of both SIRT3 overexpression and NIC administration is higher in TRAP1 knock-out cells, suggesting that targeting multiple metabolic components can dramatically hit tumor growth. Indeed, NAD^+^ levels increased the most following NIC treatment in a TRAP1-null background. This is in accord with our finding that TRAP1 ablation per se enhances complex I activity, which is suggestive of a global OXPHOS induction caused by TRAP1 inhibition [62]. Hence, the absence of TRAP1 could display its anti-tumor effect not only by inducing SDH and down-regulating the intracellular concentration of the oncometabolite succinate, but also by increasing NAD^+^/NADH ratio and the consequent SIRT3 activity via complex I induction. These data open obvious therapeutic perspectives, and the recently identified selective inhibitors of TRAP1 [49, 64, 65] are interesting candidates as antineoplastic leads in NF1-related tumors. Moreover, a higher NAD^+^/NADH ratio has widespread consequences on the metabolic equilibrium of the cell, as NAD^+^ acts as an electron acceptor in a variety of biochemical reactions that encompass glycolysis, oxidative decarboxylation of pyruvate to acetyl-CoA, β-oxidation of fatty acids and TCA cycle [66]. Therefore, further mechanisms could contribute to explain the enhanced antineoplastic efficacy of NIC administration when TRAP1 expression is knocked out.

Key findings of the present work are the identification of a complex bioenergetic adaptation in mitochondria of neurofibromin-deficient cells and the demonstration that a multiple rewiring in respiratory function contributes to the tumorigenic potential of NF1-related neoplasms. These observations constitute a conceptual starting point for drawing antineoplastic strategies based on combinatorial targeting multiple metabolic hubs in tumor models endowed with oncogenic Ras/ERK signaling.

## Supplementary information

Legends to Supplementary Figures

Supplementary Figure 1

Supplementary Figure 2

Supplementary Figure 3

Supplementary Figure 4

Supplementary Figure 5

Reproducibility Checklist Form

Original Data File

Authorship statement Masgras et al

## Data Availability

All data needed to evaluate the conclusions in the paper are present in the paper and/or the Supplementary Materials. Additional data related to this paper may be requested from the authors.
