# Supplementary material for: Tumor growth of neurofibromin-deficient cells is driven by decreased respiration and hampered by NAD+ and SIRT3
Source: Cell Death Differ. 2022 Apr 7;29(10):1996–2008. doi: 10.1038/s41418-022-00991-4 (PMC9525706; doi:10.1038/s41418-022-00991-4)
Supplement: Supplementary file 1 — Legends to Supplementary Figures [file 41418_2022_991_MOESM1_ESM.docx]

**SUPPLEMENTARY INFORMATION**

**Tumor growth of neurofibromin-deficient cells is driven by decreased respiration**

**and hampered by NAD^+^ and SIRT3**

Ionica Masgras^1,2,*^, Giuseppe Cannino^1^, Francesco Ciscato^1^, Carlos Sanchez-Martin^1^,  Fereshteh Babaei Darvishi^1,2^, Francesca Scantamburlo^1^, Marco Pizzi^3^, Alessio Menga^4^, Dolores Fregona^5^, Alessandra Castegna^4,6^ and Andrea Rasola^1,*^

^1^Department of Biomedical Sciences, University of Padova, 35131, Padova, Italy; ^2^Institute of Neuroscience, National Research Council, 35131, Padova, Italy; ^3^General Pathology and Cytopathology Unit, Department of Medicine-DIMED, University of Padova, 35128, Padova, Italy; ^4^Department of Biosciences, Biotechnologies and Biopharmaceutics, University of Bari, 70125, Bari, Italy; ^5^Department of Chemical Sciences, University of Padova, 35131, Padova, Italy; ^6^IBIOM-CNR, Institute of Biomembranes, Bioenergetics and Molecular Biotechnologies, National Research Council, 70126, Bari, Italy.

*Correspondence and requests for materials should be addressed to:

Andrea Rasola ([andrea.rasola@unipd.it](mailto:andrea.rasola@unipd.it))

Ionica Masgras ([ionica.masgras@gmail.com](mailto:ionica.masgras@gmail.com))

**Legends to Supplementary Figures**

**Supplementary Fig. 1 Protein levels and enzymatic activity of respiratory complex I inversely correlate with induction of Ras-MEK-ERK signaling. A** Spectrophotometric analysis of the NADH dehydrogenase activity of complex I (CI) in control (empty vector, EV) and K-RAS (G12D)-expressing Nf1^+/+^ MEFs. **B** WB analysis of ERK inhibition upon PD98059 treatment (40 µM, 3 days) in Nf1^+/+^ and Nf1^-/-^ MEFs. **C, D** WB analysis of OXPHOS proteins upon modulation of ERK activity by PD98059 treatment (40 µM, 3 days) in human U87 glioblastoma cells (**C**) and ipNF 04.4 plexiform neurofibroma cells (**D**). pERK1/2 indicates phosphorylated, active ERK1/2. Calnexin was used as a loading control. In **C**, **D**, NDUFS1, GRIM19, NDUFS3 and NDUFB8 were used as complex I markers; subunits α, UQCRC1/C2, COXII and SDHA/B as complex V, complex III, complex IV and complex II markers, respectively. **E** RT-PCR on mRNA levels of complex I, II and V subunits in Nf1^+/+^ and Nf1^-/-^ cells. Values were normalized for expression of beta-actin mRNA. Data are reported as mean ± SD values (n ≥ 3); ***:p<0.001; **: p<0.01; *: p<0.05 with a Student's *t* test analysis.

**Supplementary Fig. 2 Alternative NADH dehydrogenase NDI1 and NF1-GRD domain expression increase OCR in neurofibromin-deficient cells. A** Expression of pWPI empty vector or pWPI-NDI1 (EV and NDI, respectively) fused with GFP in Nf1^+/+^ and Nf1^-/-^ MEFs was revealed by assessing green fluorescence in cytofluorimetric inspections. **B** Representative OCR traces (left) and quantification of basal OCR values (right) in control (EV, empty vector) and GRD-expressing Nf1^-/-^ MEFs. The ATP synthase inhibitor oligomycin (0.8 µM), the proton uncoupler carbonyl cyanide-4-(trifluoromethoxy)phenylhydrazone (FCCP, 1 µM) and the respiratory complex I and III inhibitors rotenone (0.5 µM) and antimycin A (1 µM), respectively, were added where indicated. **C** Representative OCR traces of cells harboring either pWPI empty vector (EV) or pWPI-NDI1 (NDI). The complex I inhibitor rotenone (ROT, 50-200 nM) was added where indicated. Data are reported as mean ± SD values (n ≥ 3); ***: p<0.001; **: p<0.01 and *: p<0.05 with a Student's *t* test analysis.

**Supplementary Fig. 3. Alternative NADH dehydrogenase NDI1 protects from oxidative stress and cell death induced by complex I inhibitors. A**, **C** Analysis of mitochondrial ROS levels by MitoSOX staining in control and rotenone-treated (200 nM, 1 hour) cells. NDI: cells expressing the pWPI-NDI1 construct. **B**, **D** Cytofluorimetric assessment of cell viability following exposure to rotenone (200/500 nM) with or without the antioxidant N-acetyl cysteine (NAC, 500 µM). **E**, **G** Cytofluorimetric assessment of cell viability following exposure to AUL12 (2/4 μM, 24h) with or without NAC (500 µM). **F** Analysis of mitochondrial ROS levels by MitoSOX staining in control and AUL12-treated (4 μM, 1 hour) cells. All experiments in the Figure were carried out on Nf1^+/+^ and Nf1^-/-^ MEFs. Data are reported as mean ± SD values (n ≥ 3); ***: p<0.001; **: p<0.01 and *: p<0.05 with a Student's *t* test analysis or One-way ANOVA followed by Bonferroni post-test (A, B, C, E, F).

**Supplementary Fig. 4 NAD^+^ replenishment, NDI1 expression and SIRT3/SOD2 axis counteract neoplastic growth of neurofibromin-deficient cells. A** Spectrophotometric measurement of NAD^+^ levels. **B** Proliferation curve of Nf1^-/-^ MEFs. Statistical analysis was performed at 96 hours. **C** Effect of nicotinamide (NAM) treatment on soft agar growth of Nf1^-/-^ cells. **D** Matrigel-embedded 3D colony growth (right) of Nf1^-/-^ cells following SIRT3, SIRT4 or SIRT5 overexpression (left). Vinculin was used as a loading control. **E**, **G**, **H** SOD2 immunoprecipitation to assess acetylation of the SIRT3 target lysine 122 (Ac-K122 SOD2) following NDI1 expression (**E**), SIRT3 overexpression (**G**) and NIC/NAM treatment (**G**). **F** WB analysis of the expression level of SIRT3 and of its targets succinate dehydrogenase subunit A (SDHA) and isocitrate dehydrogenase 2 (IDH2). Calnexin and actin were used as loading controls. **I** Analysis of the effect on colony growth in soft agar (right) of SOD2 over-expression in Nf1^-/-^ MEFs (left). All experiments were carried out on Nf1^-/-^ MEFs. NDI: cells expressing the pWPI-NDI1 construct; EV: cells expressing the pWPI empty vector. SOD2: cells expressing the pFUGW-SOD2 vector; GFP: cells expressing the negative control pFUGW-GFP. Data are reported as mean ± SD values (n ≥ 3); ***: p<0.001; **: p<0.01 and *: p<0.05 with a Student's *t* test analysis.

**Supplementary Fig. 5 SIRT3 overexpression or TRAP1 ablation leads to inhibition of MPNST tumorigenesis. A** WB analysis of SIRT3 and SDHA/B protein levels in TRAP1 wild-type (sgEGFP) and knock-out (sgTRAP1) cisMPNST cells. Actin was used as a loading control. **B, C** Analysis of succinate-coenzyme Q reductase (SQR) activity of SDH (**B**) and of Matrigel colony formation (**C**) in TRAP1 wild-type and knock-out cisMPNST cells upon SIRT3 overexpression. In **A-C**, SIRT3: cisMPNST cells expressing pFUGW-SIRT3; GFP: cisMPNST cells expressing pFUGW-GFP. **D** Spectrophotometric analysis of the NADH dehydrogenase activity of respiratory complex I (CI) in wild type and TRAP1 knock out sMPNST undergoing tumorigenic growth (day 3^rd^ and 5^th^ of focus forming assay). **E** WB analysis of complex I subunits NDUFS1, NDUFS3 and NDUFB8 in wild-type and TRAP1 knock-out sMPNST cells in basal condition (monolayer growth) and undergoing tumorigenic growth (day 3^rd^, 4^th^ and 5^th^ of focus forming assay). SDHA was used as a loading control. **F** WB analysis of HIF1α upon CoCl_2_ treatment (0.5 mM, 6 hours) and colony formation of sMPNST cells upon HIF1α silencing. shSCR: scramble shRNA sequence; shHIF1α #20 and #22 are targeting two different sequences of the HIF1α coding region. **G** Colony formation of sMPNST cells upon overexpression of human wild-type or mutated P402A/P564A HIF1α. Data are reported as mean ± SD values (n ≥ 3); ***: p<0.001; **: p<0.01 and *: p<0.05 with a Student's *t* test analysis or One-way ANOVA followed by Bonferroni post-test (B, C, G).

**Tables**

**Supplementary Table 1** List of primers used for qPCR.

| **Gene** | **Forward primer** | **Reverse primer** |
| --- | --- | --- |
| Actb | CCCCCTGAACCCTAAGGCCA | GGCTACGTACATGGCTGGGG |
| Sdha | CGGCTTTCACTTCTCTGTTGGTGA | AAAGGCCAAATGCAGCTCGCAA |
| Ndufs1 | TCTTCTGGGAGCAGATGGAGGT | ATGGGAGCACCAACATCACCA |
| Ndufs3 | ATGGCTTCGAGGGACATCCT | GGTTCAGCCACTACCCGCTT |
| Grim19 | CTACGGCCCCATCGACTACAAG | CCCCGATGCCCACAGCAAAC |
| ATP5a1 | GGCTGGTGATGTGTCCGCTT | TTTGGGCAGCAGATCCGACA |

**Supplementary Table 2** List of antibodies used for WB and IHC analysis.

| Mouse monoclonal anti-human TRAP1 (clone TR-1A) | Santa Cruz Biotechnology | Cat#sc-73604, RRID: AB_1130629 |
| --- | --- | --- |
| Mouse monoclonal anti-rodent TRAP1 (clone 42) | Becton Dickinson | Cat#612344, RRID: AB_399710 |
| Mouse monoclonal anti-SDHA (clone D-4) | Santa Cruz Biotechnology | Cat#sc-166947, RRID: AB_10610526 |
| Mouse monoclonal anti-β ACTIN (clone C4) | Santa Cruz Biotechnology | Cat#sc-47778, RRID: AB_2714189 |
| Rabbit polyclonal anti-HIF-1α | Novus Biologicals | Cat# NB100-449, RRID:AB_10001045 |
| Rabbit monoclonal anti-NDUFS1 | Abcam | Cat# ab157221, RRID:AB_2857900 |
| Mouse monoclonal OXPHOS antibody cocktail recognizing CI (NDUFB8), CII (SDHB), CIII (UQCRC2), CIV (COXII) and CV (α) | Abcam | Cat# ab110411, RRID:AB_2756818 |
| Mouse monoclonal anti-NDUFS3 antibody | Thermo Fisher Scientific | Cat# 459130, RRID:AB_2532226 |
| Mouse monoclonal anti-GRIM19 | Santa Cruz Biotechnology | Cat# sc-271013, RRID:AB_10612900 |
| Mouse monoclonal anti-UQCRC1 | Santa Cruz Biotechnology | Cat# sc-65238, RRID:AB_2213631 |
| Goat polyclonal anti-CALNEXIN | Santa Cruz Biotechnology | Cat# sc-6465, RRID:AB_2069146 |
| Rabbit monoclonal anti-ERK1/2 | Cell Signaling | Cat# 4695, RRID:AB_390779 |
| Rabbit polyclonal anti-phosphor-ERK1/2 | Cell Signaling | Cat# 9101, RRID:AB_331646 |
| Rabbit anti-NDI1 | Gift of T. Yagi | Laboratory of Eric Dufour |
| Rabbit monoclonal anti-SIRT3 | Cell Signaling | Cat# 5490, RRID:AB_10828246 |
| Rabbit polyclonal anti-SOD2 | Abcam | Cat# ab13534, RRID:AB_2191667 |
| Rabbit monoclonal anti-Acetyl-K122 SOD2 | Cell Signaling | ab214675 |
| Mouse monoclonal anti-IDH2 | Abcam | Cat# ab55271, RRID:AB_943793 |
| Mouse monoclonal anti-k-RAS | Santa Cruz Biotechnology | Cat# sc-30, RRID:AB_627865 |
| Mouse monoclonal anti-CypD | Calbiochem | Cat# AP1035, RRID:AB_2169442 |
