# Supplementary figures and images for: Tumor growth of neurofibromin-deficient cells is driven by decreased respiration and hampered by NAD+ and SIRT3
Source: Cell Death Differ. 2022 Apr 7;29(10):1996–2008. doi: 10.1038/s41418-022-00991-4 (PMC9525706; doi:10.1038/s41418-022-00991-4)

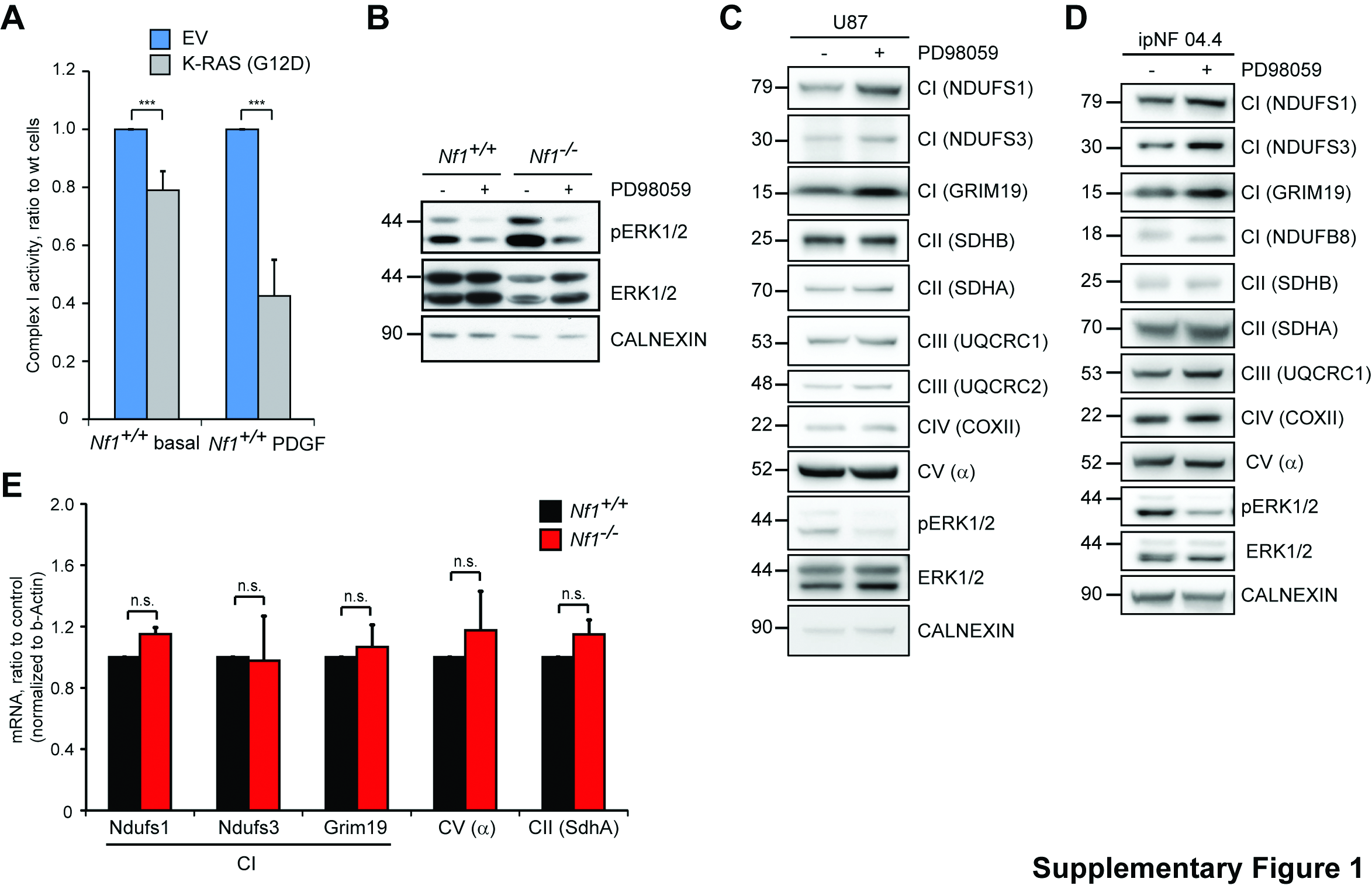

Supplement: Supplementary file 2 — Supplementary Figure 1 [file 41418_2022_991_MOESM2_ESM.tif]

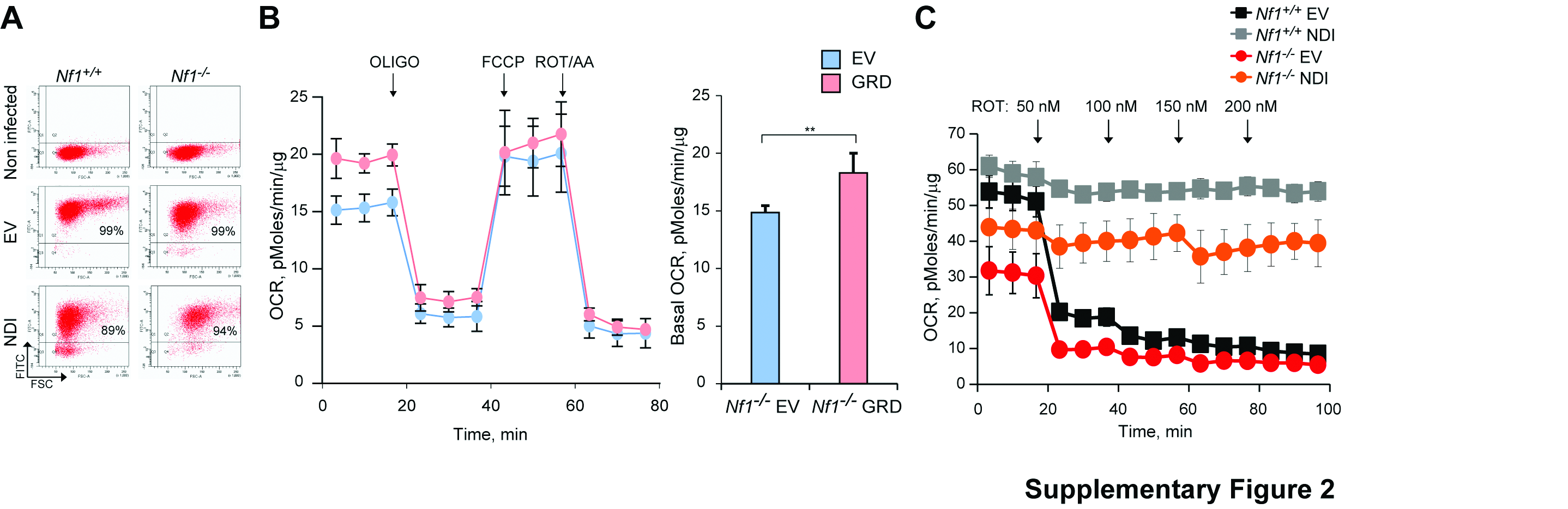

Supplement: Supplementary file 3 — Supplementary Figure 2 [file 41418_2022_991_MOESM3_ESM.tif]

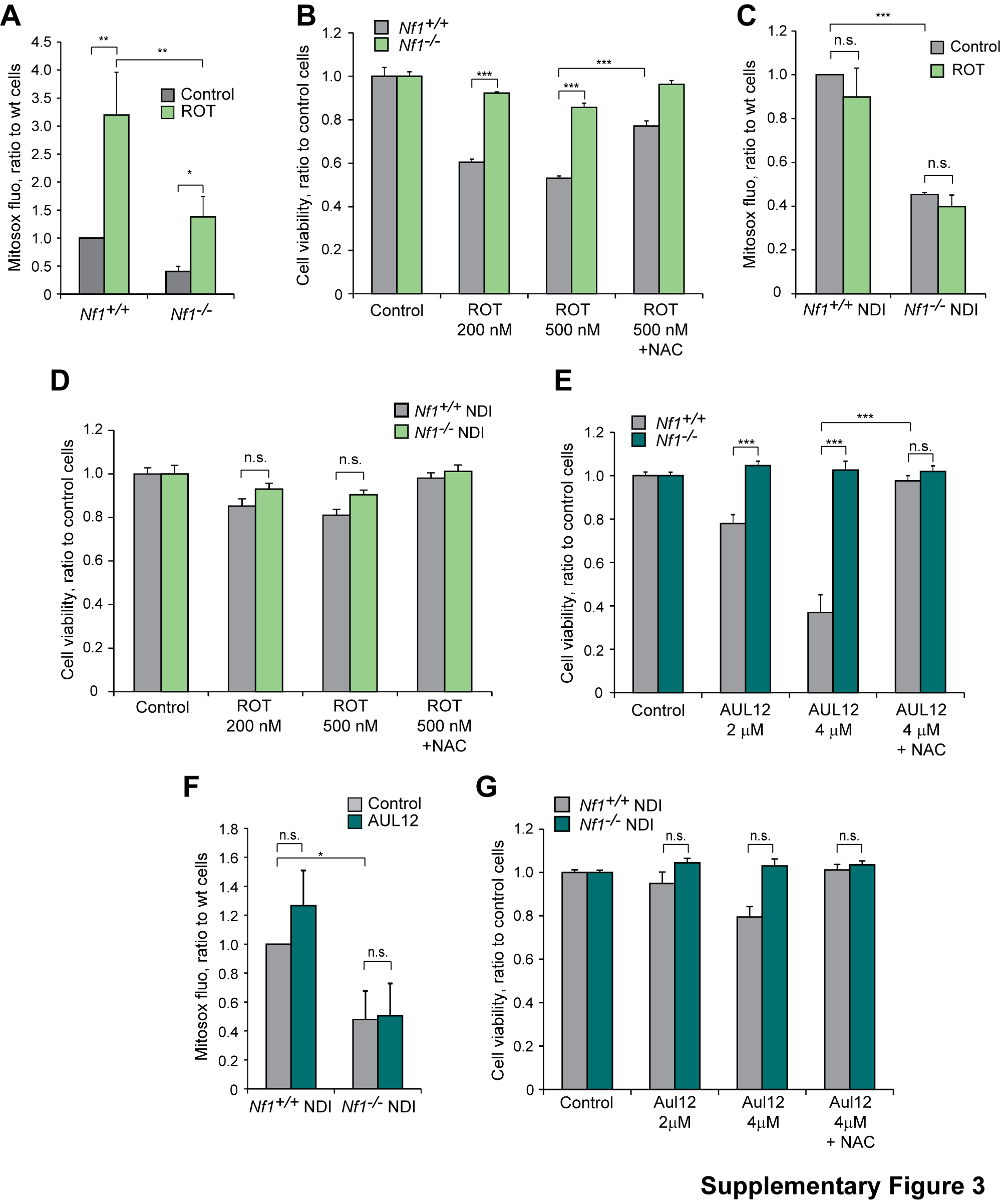

Supplement: Supplementary file 4 — Supplementary Figure 3 [file 41418_2022_991_MOESM4_ESM.tif]

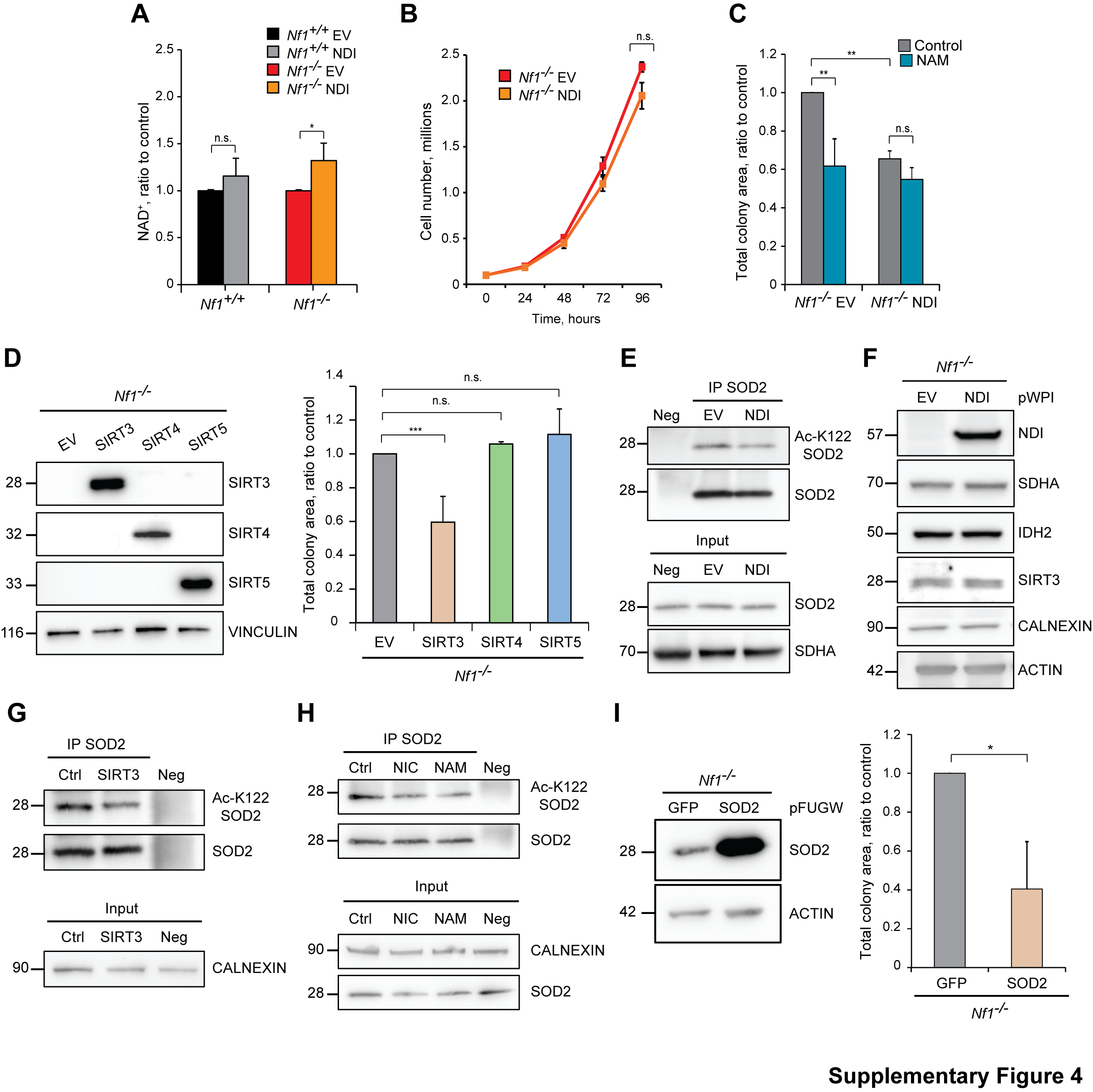

Supplement: Supplementary file 5 — Supplementary Figure 4 [file 41418_2022_991_MOESM5_ESM.tif]

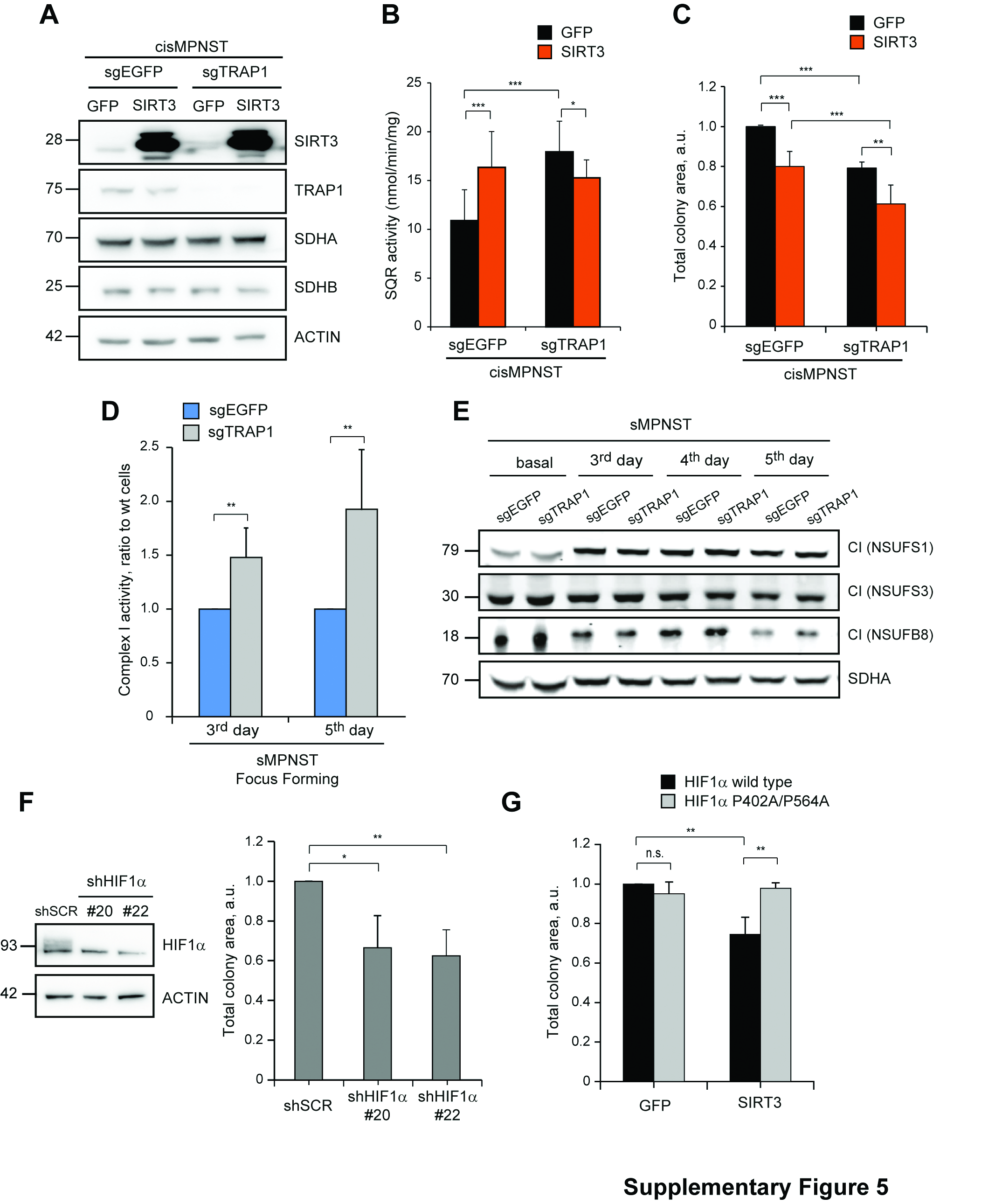

Supplement: Supplementary file 6 — Supplementary Figure 5 [file 41418_2022_991_MOESM6_ESM.tif]
