# Supplementary material for: Tumor growth of neurofibromin-deficient cells is driven by decreased respiration and hampered by NAD+ and SIRT3
Source: Cell Death Differ. 2022 Apr 7;29(10):1996–2008. doi: 10.1038/s41418-022-00991-4 (PMC9525706; doi:10.1038/s41418-022-00991-4)
Supplement: Supplementary file 8 — Original Data File [file 41418_2022_991_MOESM8_ESM.pdf]

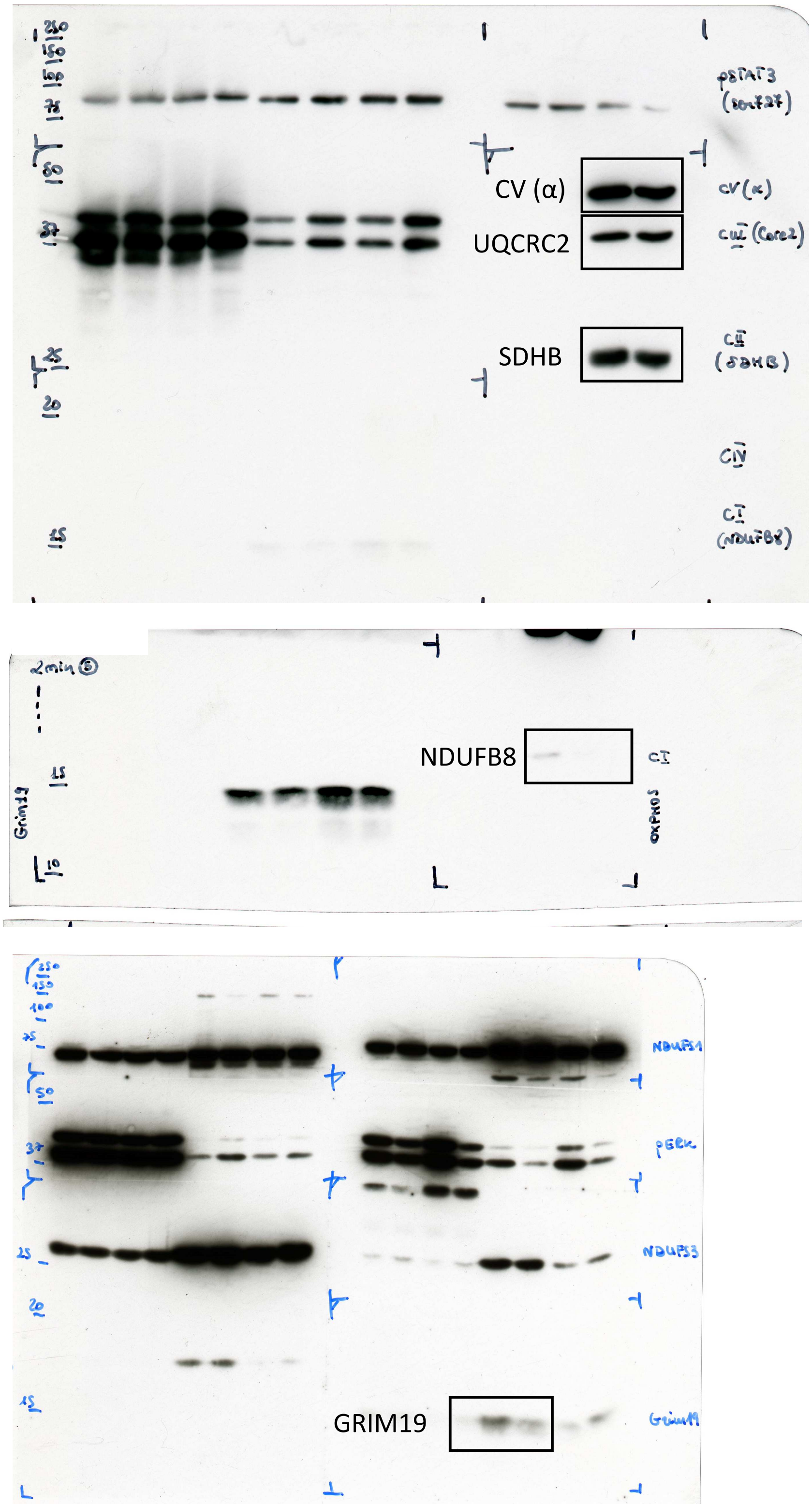

Fig. 1A

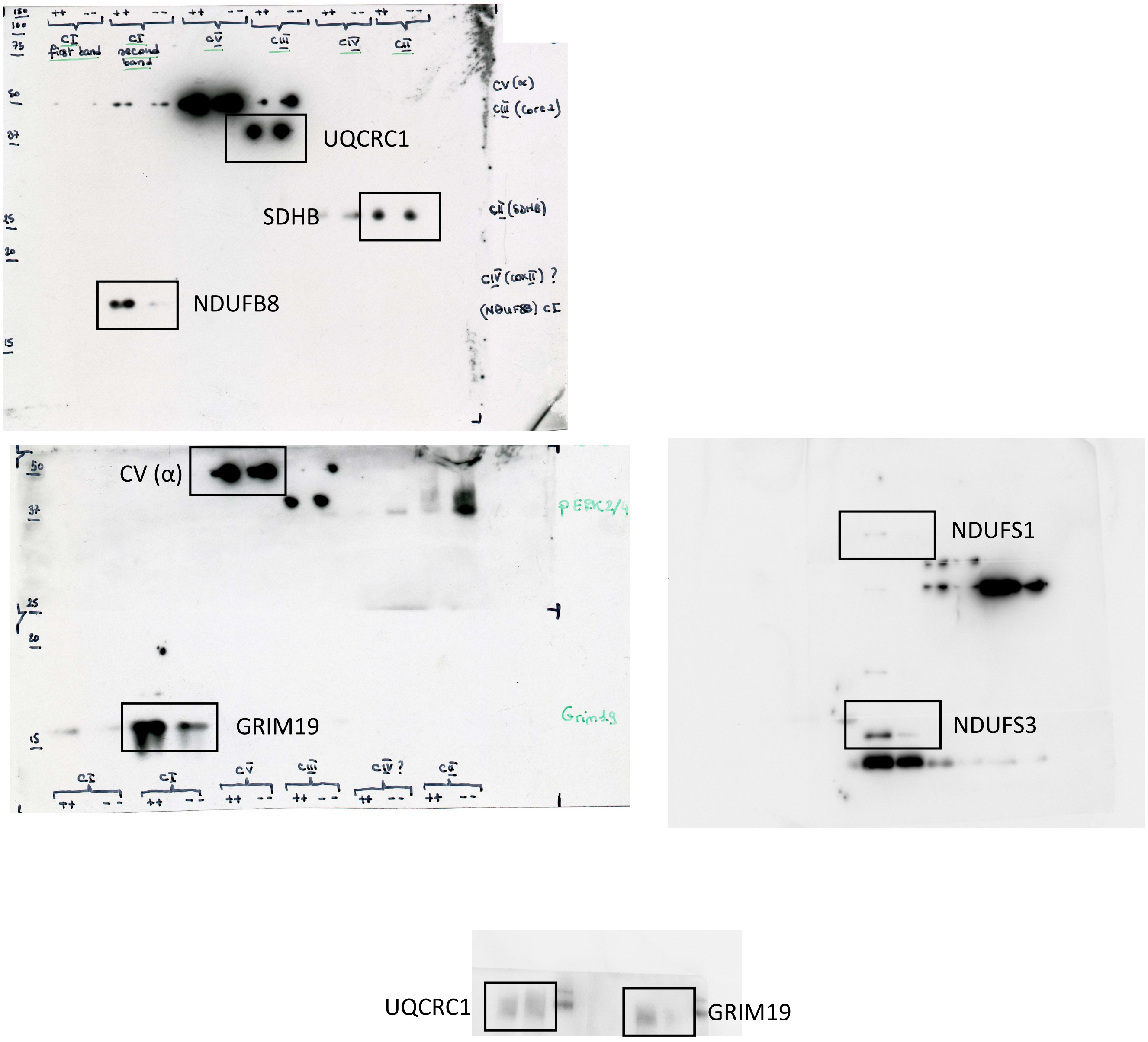

Fig. 1B, D

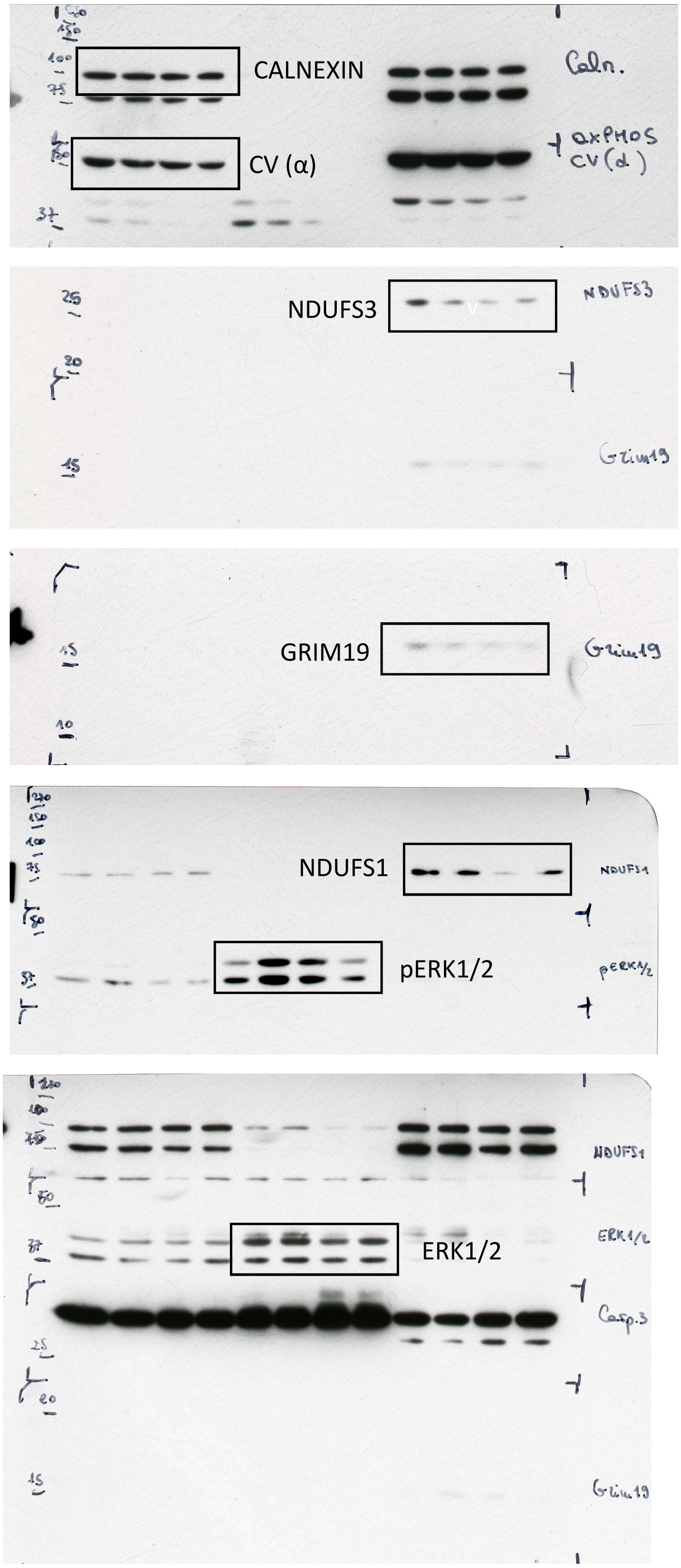

Fig. 2A

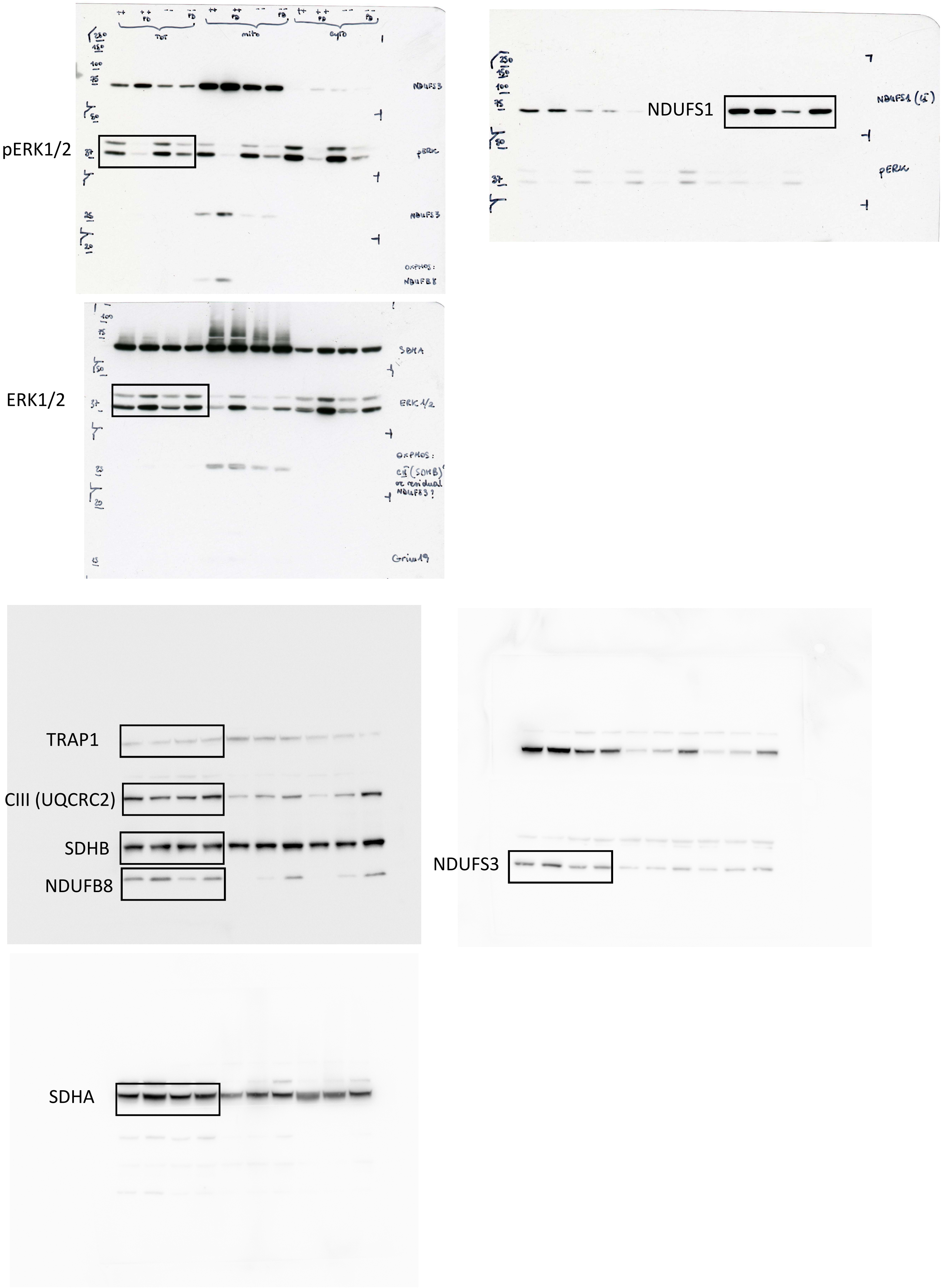
pERK1/2
NDUFS1
ERK1/2
TRAP1
CIII (UQCRC2)
SDHB
NDUFB8
NDUFS3
SDHA

Fig. 2B

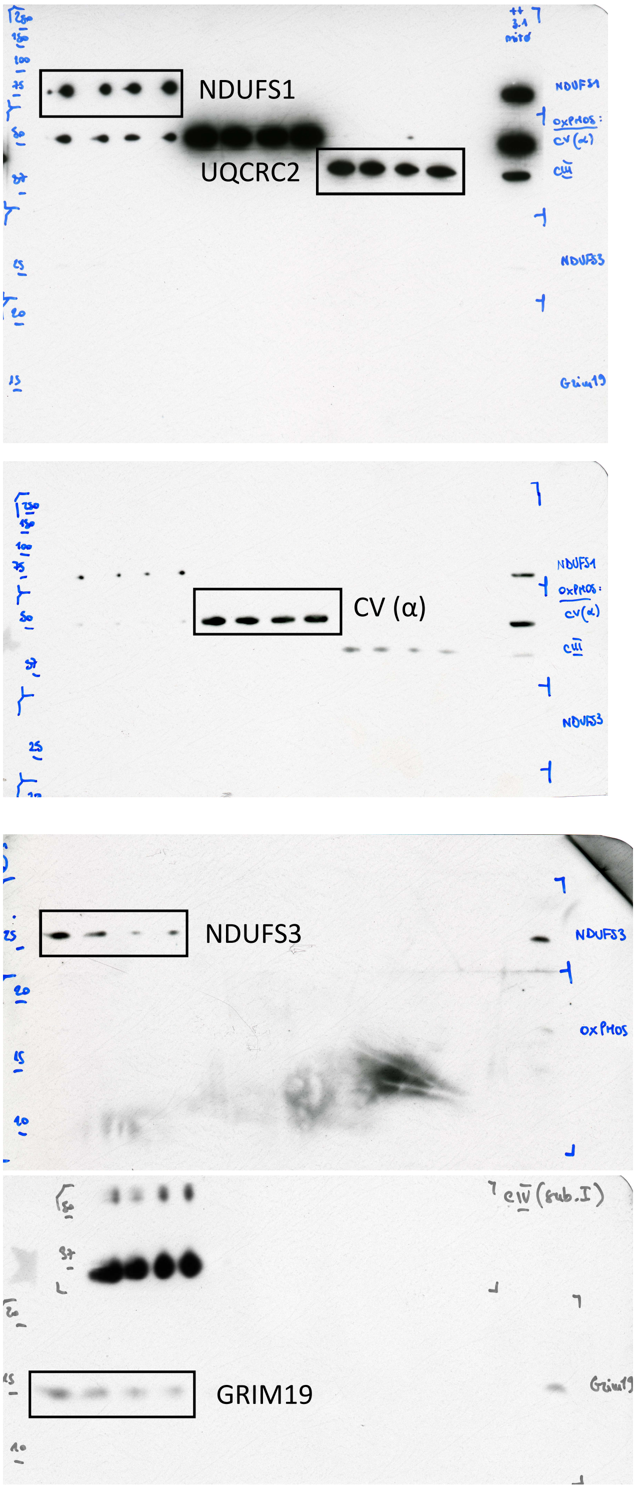

Fig. 2D

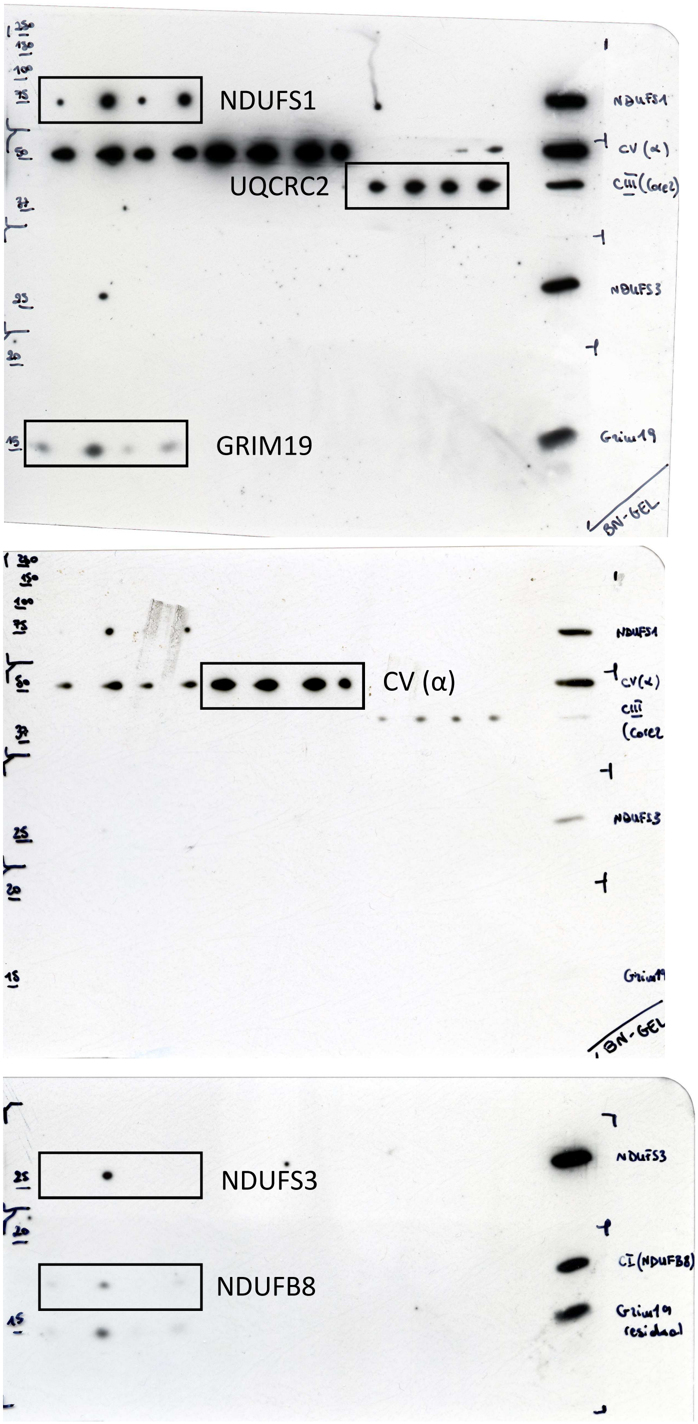

Fig. 2E

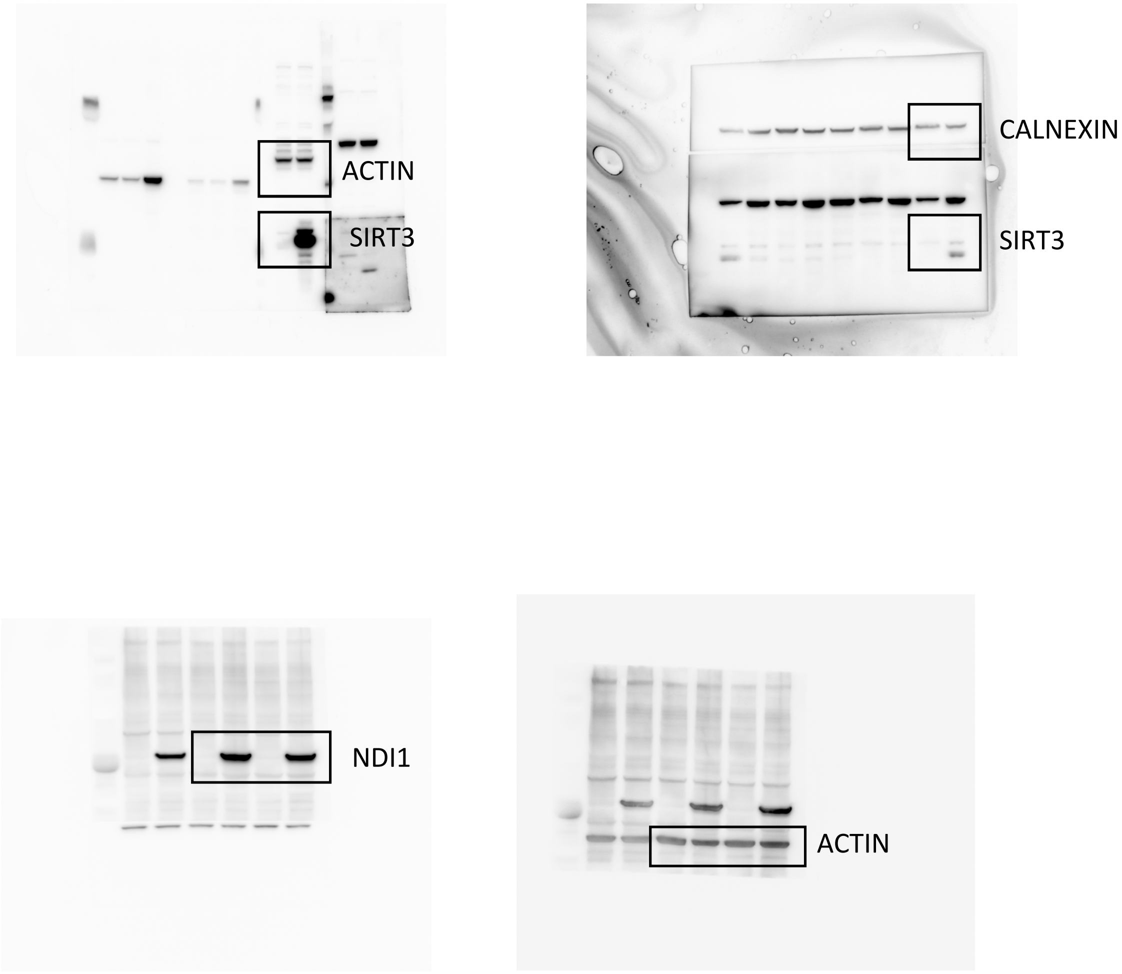

Fig. 4

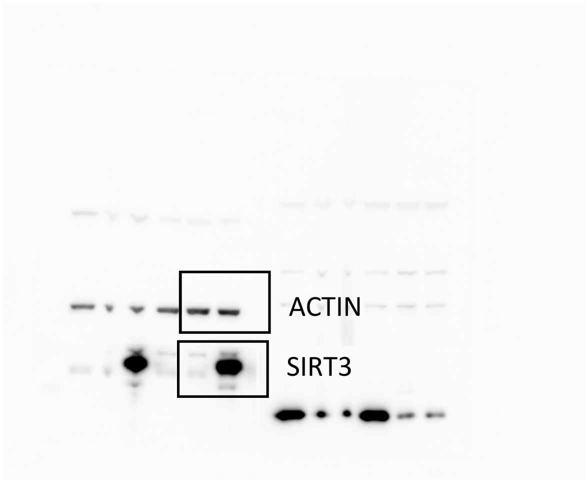

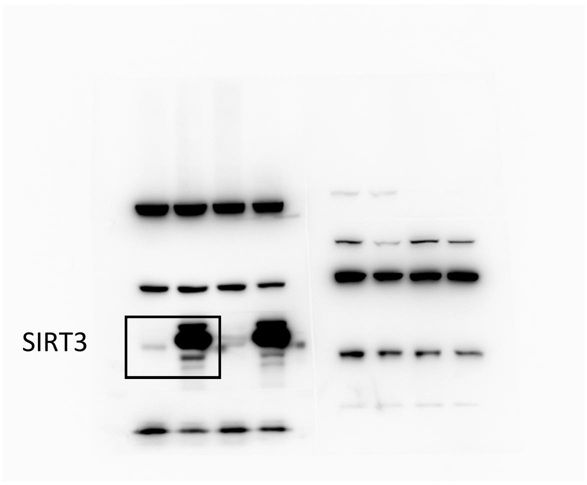

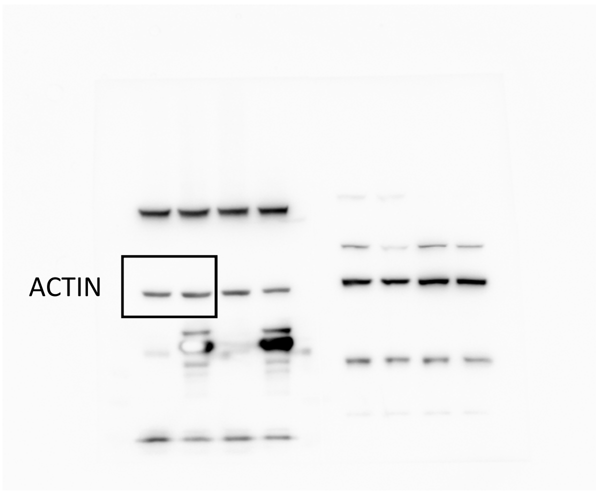

Fig. 5

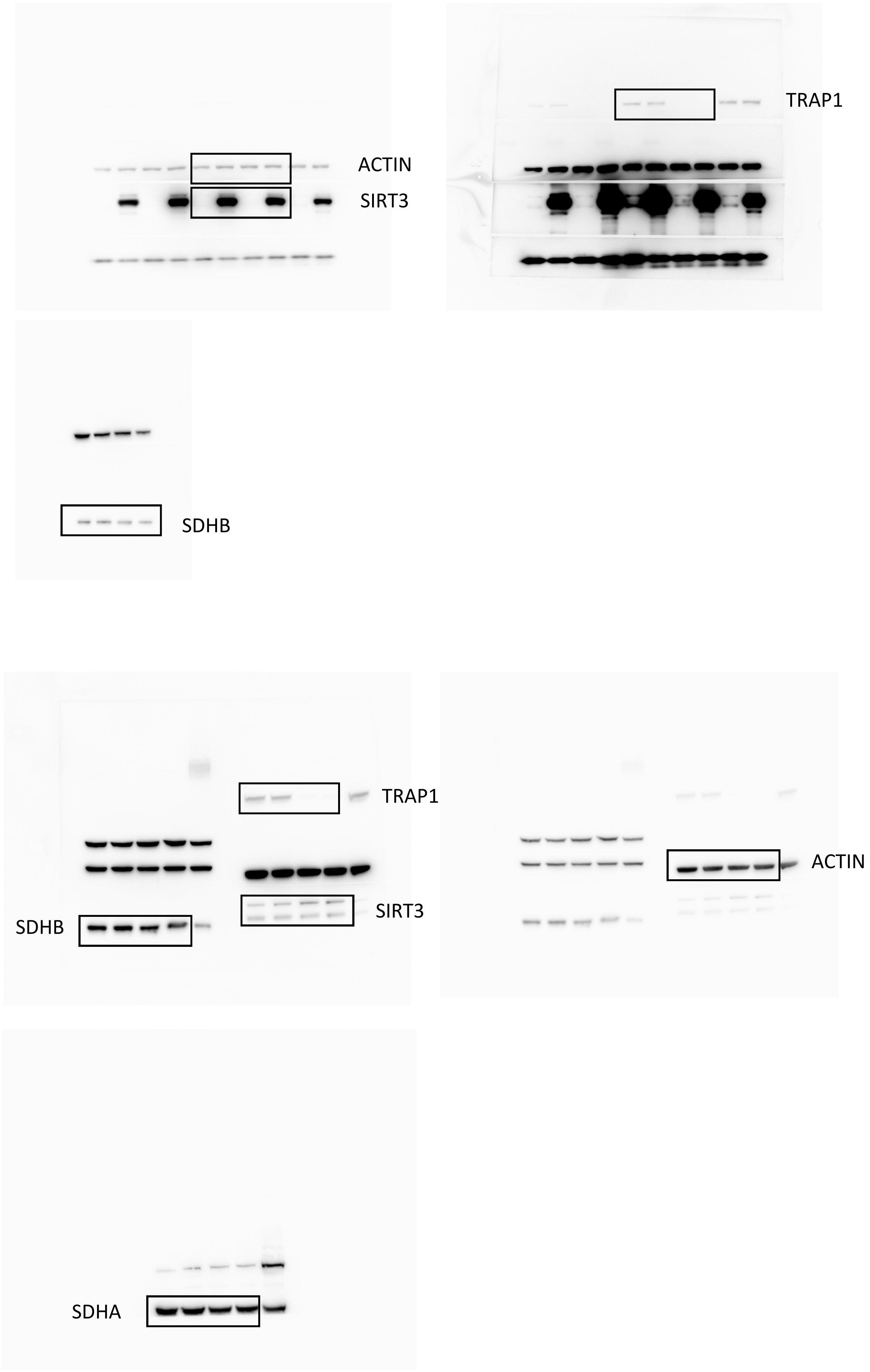

Fig. 6

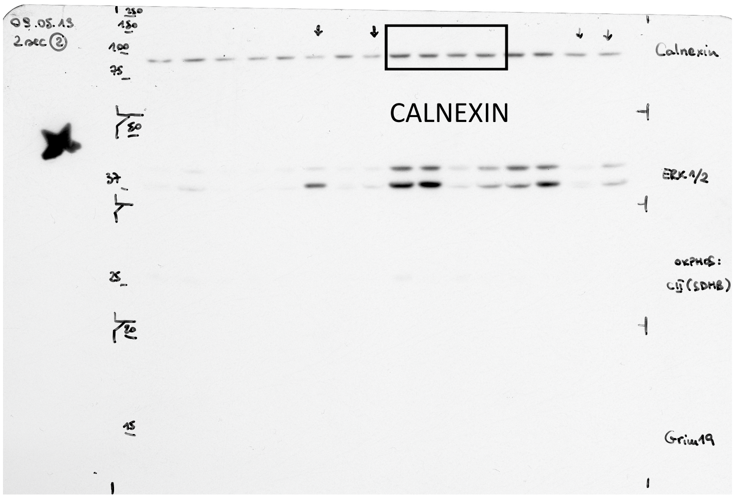

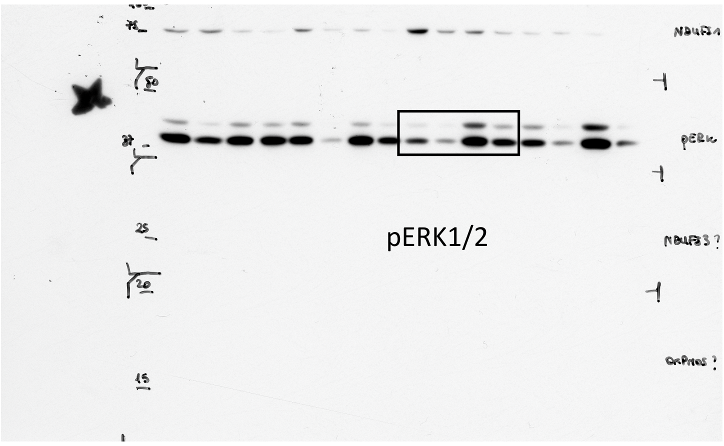

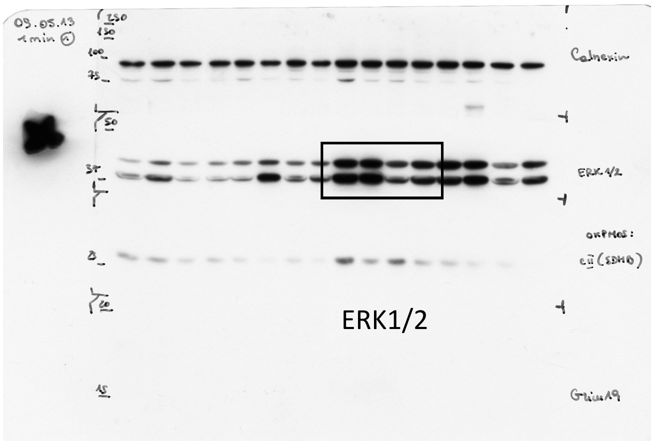

Supplementary Fig. 1B

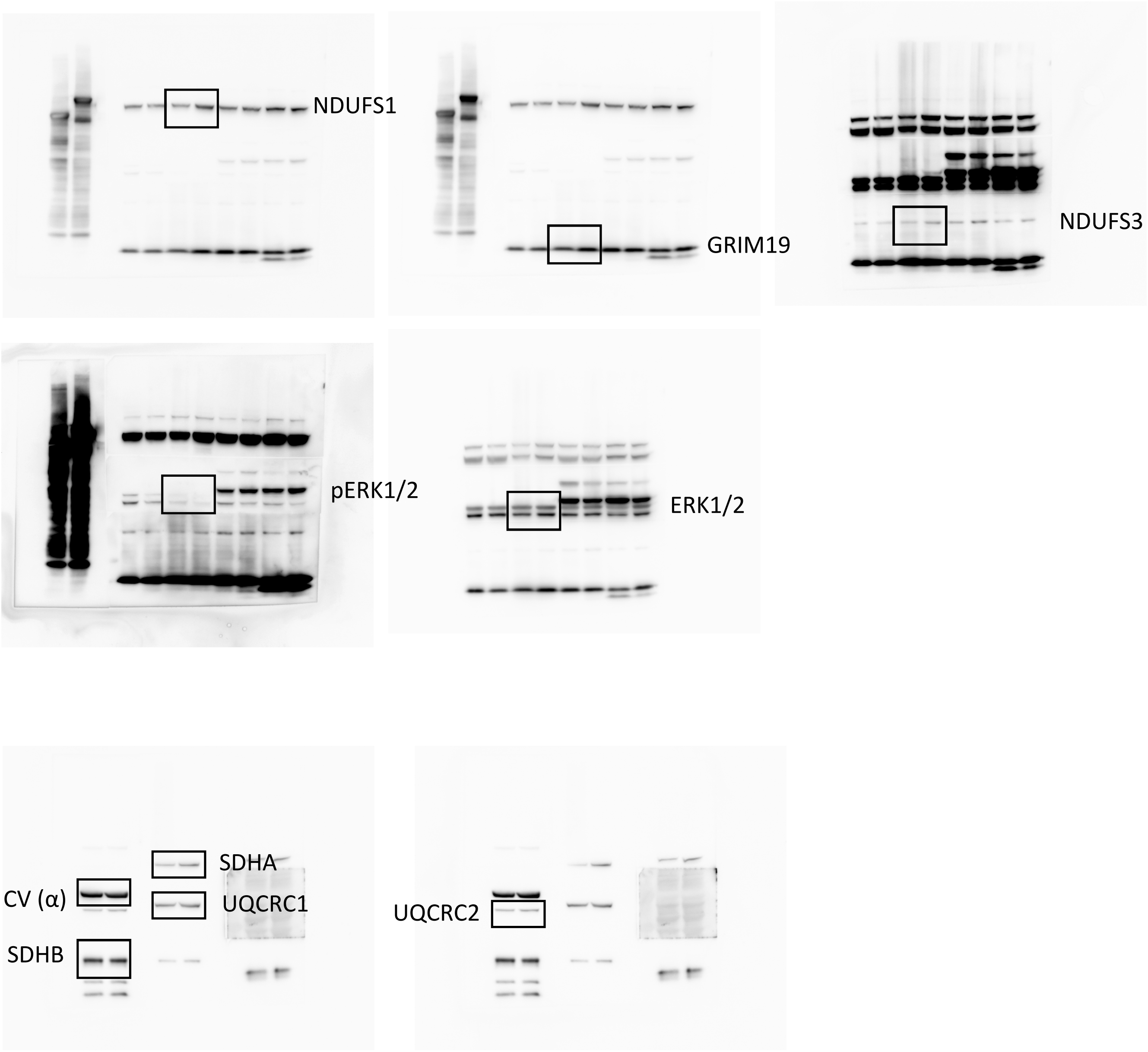

Supplementary Fig. 1C

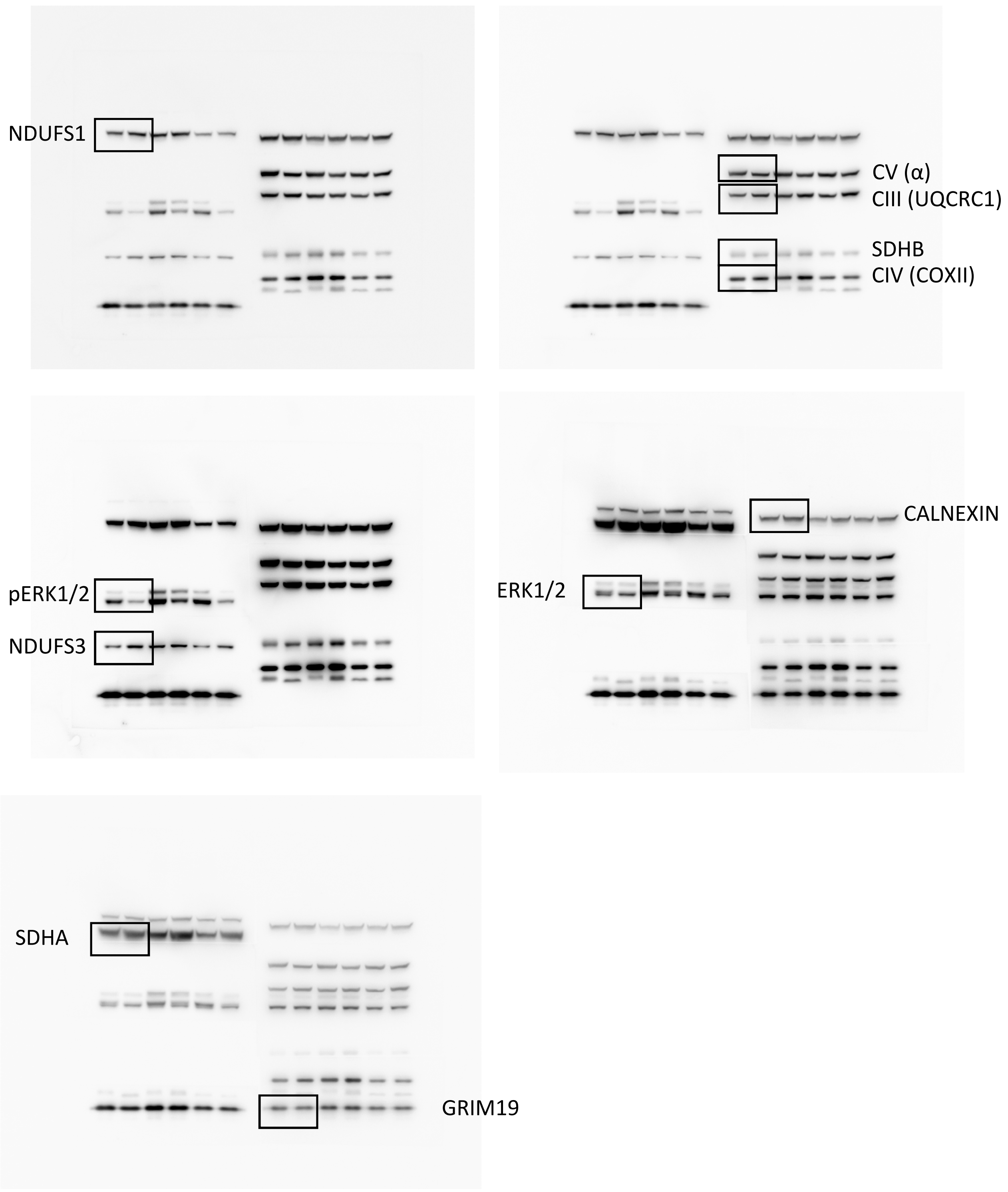

Supplementary Fig. 1D

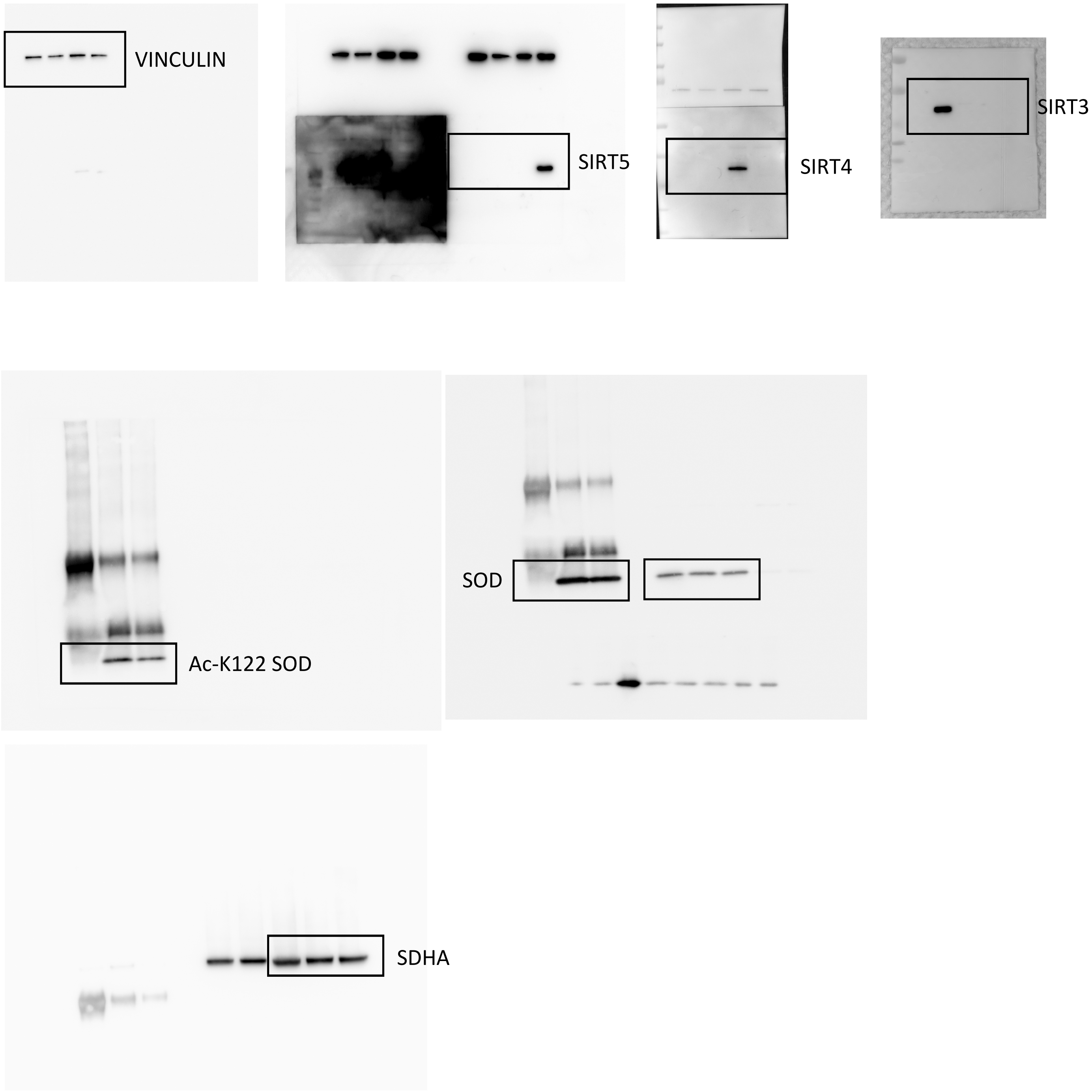

Supplementary Fig. 4D, E

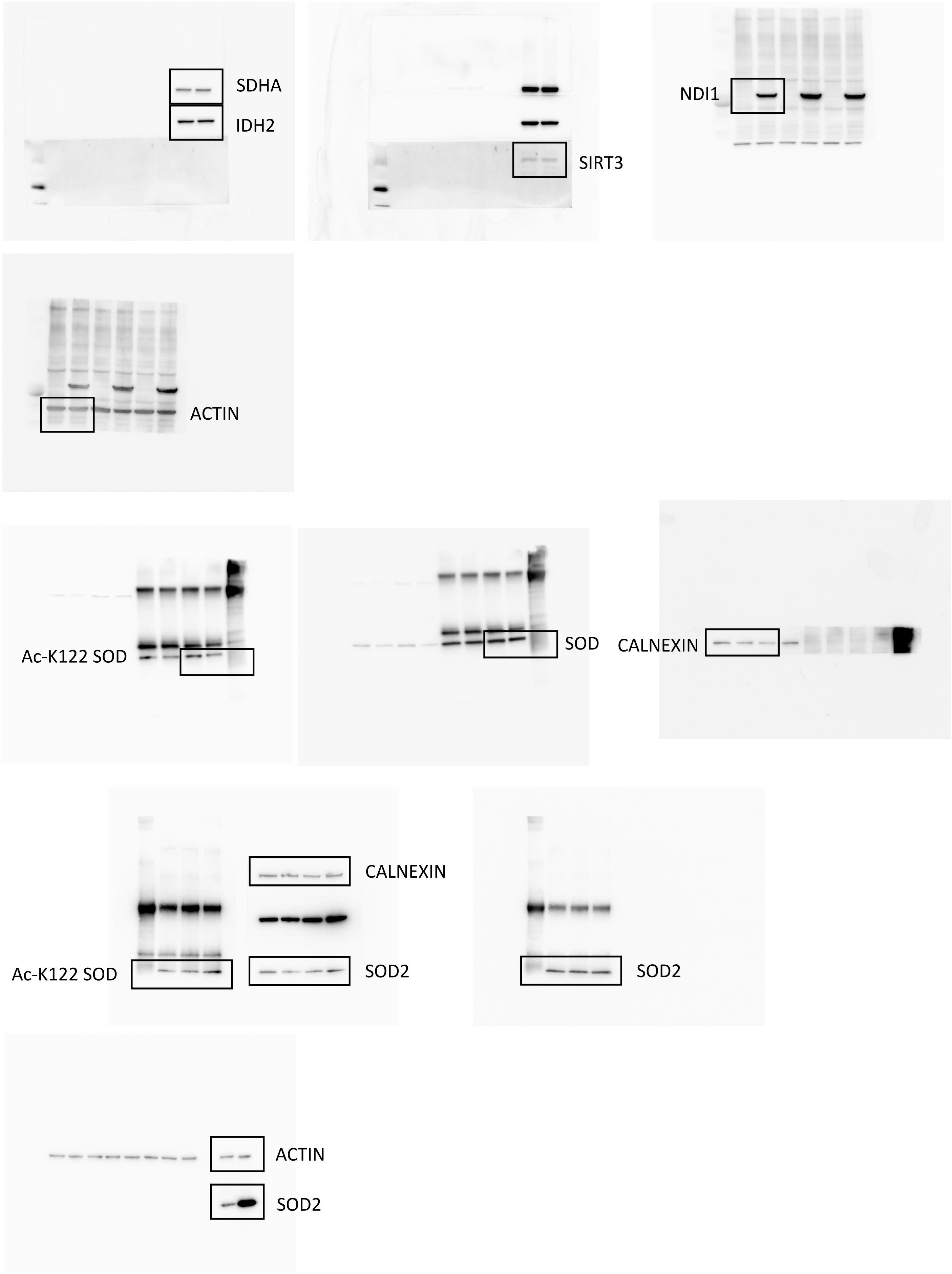

Supplementary Fig. 4F-I

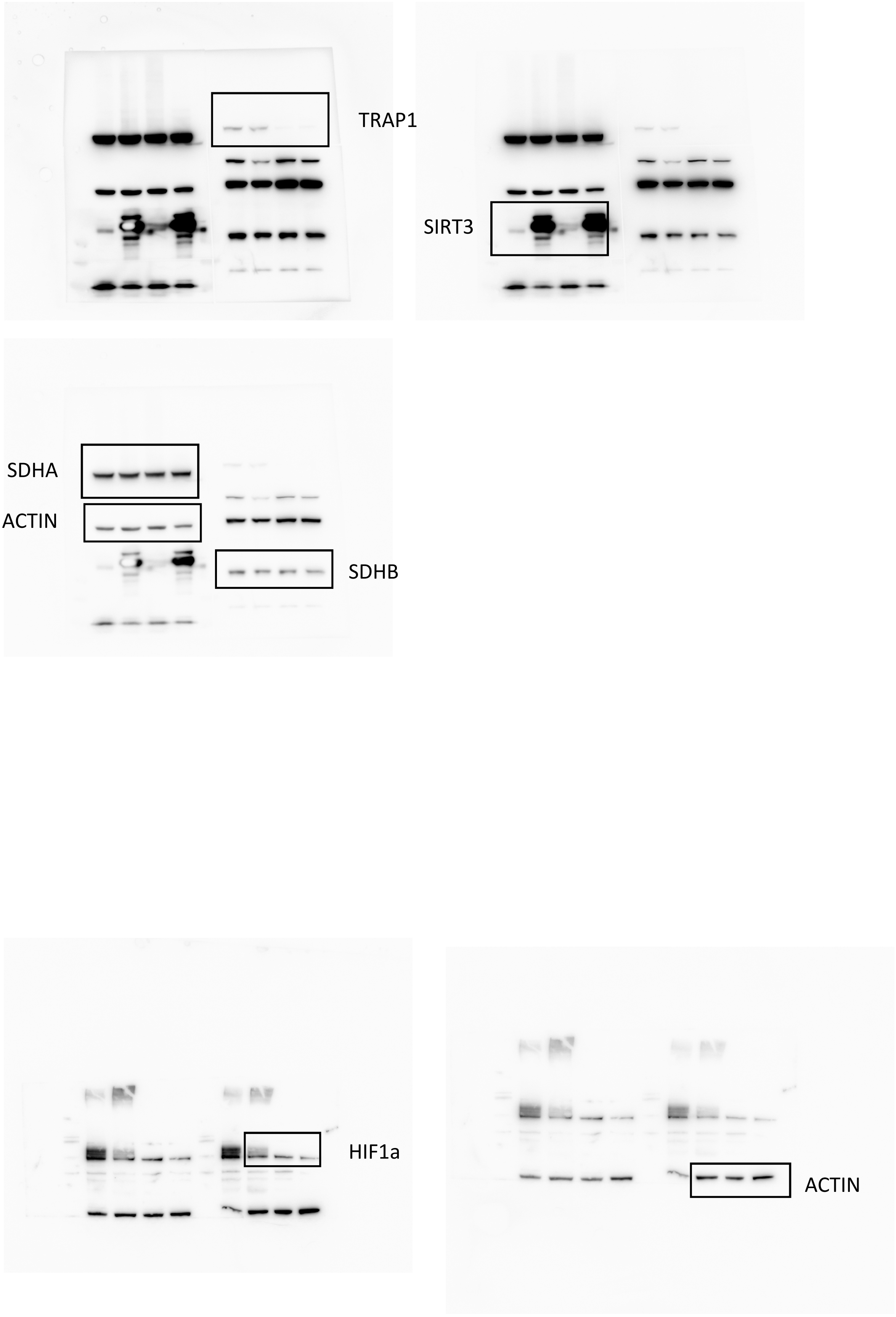

Supplementary Fig. 5A, F

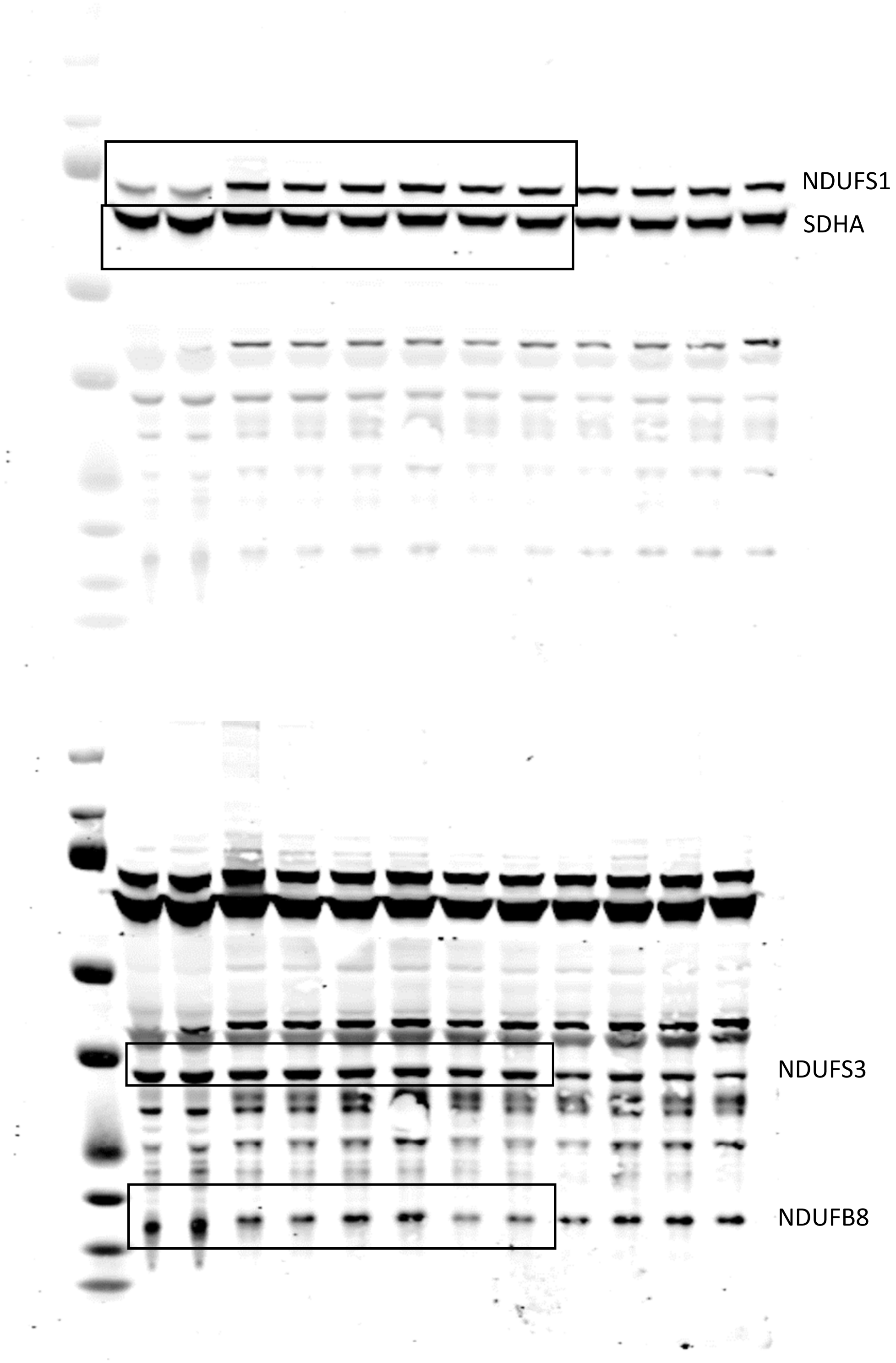

Supplementary Fig. 5E
