# Supplementary material for: Tumor growth of neurofibromin-deficient cells is driven by decreased respiration and hampered by NAD+ and SIRT3
Source: Cell Death Differ. 2022 Apr 7;29(10):1996–2008. doi: 10.1038/s41418-022-00991-4 (PMC9525706; doi:10.1038/s41418-022-00991-4)
Supplement: Supplementary file 9 — Authorship statement Masgras et al [file 41418_2022_991_MOESM9_ESM.pdf]

SPRINGER NATURE

Change of authorship request form - Journals (pre-acceptance)

Important information. Please read.

- This form should be used by authors to request any change in authorship (adding/deleting authors) including changes in corresponding authors. This form should not be used for name changes. Please fully complete all sections. Use black ink and block capitals and provide each author's full name with the given name first followed by the family name.

- By signing this declaration, all authors guarantee that the order of the authors are in accordance with their scientific contribution, if applicable as different conventions apply per discipline, and that only authors have been added who made a meaningful contribution to the work.

- Please note, in author collaborations where there is formal agreement for representing the collaboration, it is sufficient for the representative or legal guarantor (usually the corresponding author) to complete and sign the Authorship Change Form on behalf of all authors, **next to the added/removed author(s)**. **(Complete Section 3, followed by Section 6.)**
  In author collaborations where there is no formal agreement for representing the collaboration and **there are more than 10 authors,** one may sign for all, provided the signer appends correspondence that attests that each of the authors have agreed to the change **and the added/removed authors sign the form**. **(Complete Section 3, followed by Section 6.)**

- Please note, we cannot investigate or mediate any authorship disputes. If you are unable to obtain agreement from all authors (including those who you wish to be removed) you must refer the matter to your institution(s) for investigation. Please inform us if you need to do this.

- If you are not able to return a fully completed form within **30 days** of the date that it was sent to the author requesting the change, we may have to withdraw your manuscript. We cannot publish manuscripts where authorship has not been agreed by all authors (including those who have been removed).

- Incomplete forms will be rejected.

- Please return/upload this form, fully completed, to the Journals Editorial Office. The Journal and/or Publisher will consider the information you have provided to decide whether to approve the proposed change in authorship. We may decide to contact your institution for more information or undertake a further investigation, if appropriate, before making a final decision.

SPRINGER NATURE

Change of authorship request form - Journals (pre-acceptance)

Section 1: Please provide the current title of manuscript

Manuscript ID no.: CDD-21-1654

Title: TUMOR GROWTH OF NEUROFIBROMIN-DEFICIENT CELLS IS DRIVEN BY DECREASED RESPIRATION AND HAMPERED BY NAD+ AND SIRT3

Section 2: Please provide the previous authorship, in the order shown on the manuscript before the changes were introduced. Please indicate the corresponding author by adding (CA) behind the name.

	First name(s)	Family name	ORCID or SCOPUS id, if available
1st author	IONICA	MASGRAS	0000-0002-9768-460X
2nd author	GIUSEPPE	CANNINO	
3rd author	FRANCESCO	CISCATO	
4th author	CARLOS	SANCHEZ-MARTIN	
5th author	MARCO	PIZZI	0000-0003-4006-7317
6th author	ALESSIO	MENGA	
7th author	DOLORES	FREGONA	0000-0002-8101-1101
8th author	ALESSANDRA	CASTEGNA	
9th author	ANDREA	RASOLA	0000-0003-4522-3008
10th author			

Please use an additional sheet if there are more than 10 authors.

SPRINGER NATURE Springer Nature is one of the world's leading global research, educational and professional publishers, created in May 2015 through the combination of Nature Publishing Group, Palgrave Macmillan, Macmillan Education and Springer Science+Business Media.

SPRINGER NATURE

Change of authorship request form - Journals (pre-acceptance)

Section 3: Please provide a justification for change. Please use this section to explain your reasons for changing the authorship of your manuscript, e.g. what necessitated the change in authorship? Please refer to the (journal) policy pages for more information about authorship. Please explain why omitted authors were not originally included and/or why authors were removed on the submitted manuscript.

WE INCLUDED 2 ADDITIONAL AUTHORS FOR THIS MANUSCRIPT AS THEY CONTRIBUTED TO THE REVISION PROCESS.

Section 4: Proposed new authorship. Please provide your new authorship list in the order you would like it to appear on the manuscript. Please indicate the corresponding author by adding (CA) behind the name. If the Corresponding Author has changed, please indicate the reason under section 3.

	First name(s)	Family name (this name will appear in full on the final publication and will be searchable in various abstract and indexing databases)	Affiliated institute	E-mail address
1st author	IONICA	MASGRAS	CNR; UNIVERSITY OF PADOVA	ionica.masgras@gmail.com
2nd author	GIUSEPPE	CANNINO	UNIVERSITY OF PADOVA	gcann1@libero.it
3rd author	FRANCESCO	CISCATO	UNIVERSITY OF PADOVA	francesco.ciscato@gmail.com
4th author	CARLOS	SANCHEZ-MARTIN	UNIVERSITY OF PADOVA	carlossanmarti@gmail.com
5th author	FERESHTEH	BABAEI DARVISHI	CNR; UNIVERSITY OF PADOVA	fereshteh.babaeidarvishi@studenti.unipd.it
6th author	FRANCESCA	SCANTAMBURLO	UNIVERSITY OF PADOVA	francescascantamburlo39@gmail.com
7th author	MARCO	PIZZI	UNIVERSITY OF PADOVA	marcopizzi2002@yahoo.it
8th author	ALESSIO	MENGA	UNIVERSITY OF BARI	mengaalessio@gmail.com
9th author	DOLORES	FREGONA	UNIVERSITY OF PADOVA	dolores.fregona@unipd.it
10th author	ALESSANDRA	CASTEGNA	CNR; UNIVERSITY OF PADOVA	acastegna@gmail.com

Please use an additional sheet if there are more than 10 authors.

SPRINGER NATURE Springer Nature is one of the world's leading global research, educational and professional publishers, created in May 2015 through the combination of Nature Publishing Group, Palgrave Macmillan, Macmillan Education and Springer Science+Business Media.

SPRINGER NATURE

Change of authorship request form - Journals (pre-acceptance)

Section 3: Please provide a justification for change. Please use this section to explain your reasons for changing the authorship of your manuscript, e.g. what necessitated the change in authorship? Please refer to the (journal) policy pages for more information about authorship. Please explain why omitted authors were not originally included and/or why authors were removed on the submitted manuscript.

/

Section 4: Proposed new authorship. Please provide your new authorship list in the order you would like it to appear on the manuscript. Please indicate the corresponding author by adding (CA) behind the name. If the Corresponding Author has changed, please indicate the reason under section 3.

	First name(s)	Family name (this name will appear in full on the final publication and will be searchable in various abstract and indexing databases)	Affiliated institute	E-mail address
1st author	ANDREA	RASOLA	UNIVERSITY OF PADOVA	andrea.rasola@unipd.it
2nd author				
3rd author				
4th author				
5th author				
6th author				
7th author				
8th author				
9th author				
10th author				

Please use an additional sheet if there are more than 10 authors.

SPRINGER NATURE Springer Nature is one of the world's leading global research, educational and professional publishers, created in May 2015 through the combination of Nature Publishing Group, Palgrave Macmillan, Macmillan Education and Springer Science+Business Media.

SPRINGER NATURE

Change of authorship request form - Journals (pre-acceptance)

Section 5: Author contribution, Acknowledgement and Disclosures. Please use this section to provide a new disclosure statement and, if appropriate, acknowledge any contributors who have been removed as authors and ensure you state what contribution any new authors made (if applicable per the journal or book (series) policy). Please ensure these are updated in your manuscript - after approval of the change(s) - as our production department will not transfer the information in this form to your manuscript.

New acknowledgements: NOT APPLICABLE

New Disclosures (financial and non-financial interests, funding): NOT APPLICABLE

New Author Contributions statement (if applicable per the journal policy): INVESTIGATION: F.B.D and F.S. have been added to the original list of authors.

State 'Not applicable' if there are no new authors.

SPRINGER NATURE Springer Nature is one of the world's leading global research, educational and professional publishers, created in May 2015 through the combination of Nature Publishing Group, Palgrave Macmillan, Macmillan Education and Springer Science+Business Media.

SPRINGER NATURE

Change of authorship request form - Journals (pre-acceptance)

Section 6: Declaration of agreement. ***All* authors, unchanged, new and removed *must* sign this declaration.**
(NB: Please print the form, (docu)-sign and return/upload a scanned copy. Please note that signatures that have been inserted as an image file are acceptable as long as it is handwritten. Typed names in the signature box are unacceptable.) *** Please delete as appropriate. Delete all of the bold if you were on the original authorship list and are remaining as an author.**

	First name	Family name		Signature	Date
1st author	IONICA	MASGRAS	I agree to the proposed new authorship shown in section 4 **/and the addition~~/removal*of my~~ name to the authorship list** ~~/and the proposed change in corresponding author~~	Ionica Masgras	17/03/22
2nd author	GIUSEPPE	CANNINO	I agree to the proposed new authorship shown in section 4 **/and the addition/~~removal*of my~~ name to the authorship list** ~~/and the proposed change in corresponding author~~		
3rd author	FRANCESCO	CISCATO	I agree to the proposed new authorship shown in section 4 **/and the addition/~~removal*of my~~ name to the authorship list** ~~/and the proposed change in corresponding author~~	Francesco Ciscato	17/03/22
4th authors	CARLOS	SANCHEZ-MARTIN	I agree to the proposed new authorship shown in section 4 **/and the addition/~~removal*of my~~ name to the authorship list** ~~/and the proposed change in corresponding~~ author		
5th author	FERESHTEH	BABAEI DARVISHI	I agree to the proposed new authorship shown in section 4 **/and the addition/~~removal~~*of my name to the authorship list** ~~/and the proposed change in corresponding author~~	[illegible]	17.03.2022
6th author	FRANCESCA	SCANTAMBURLO	I agree to the proposed new authorship shown in section 4 **/and the addition~~/removal~~*of my name to the authorship list** ~~/and the proposed change in corresponding author~~	Francesca Scantamburlo	17/03/22
7th author	MARCO	PIZZI	I agree to the proposed new authorship shown in section 4 **/and the addition/~~removal*of my~~ name to the authorship list** ~~/and the proposed change in corresponding author~~		

SPRINGER NATURE Springer Nature is one of the world's leading global research, educational and professional publishers, created in May 2015 through the combination of Nature Publishing Group, Palgrave Macmillan, Macmillan Education and Springer Science+Business Media.

Change of authorship request form - Journals (pre-acceptance)

	First name	Family name		Signature	Date
8th author	ALESSIO	MENGA	I agree to the proposed new authorship shown in section 4 **/and the addition/~~removal*~~of my name to the authorship list** /~~and the proposed change in corresponding author~~		
9th author	DOLORES	FREGONA	I agree to the proposed new authorship shown in section 4 **/and the addition/~~removal*~~of my name to the authorship list** /~~and the proposed change in corresponding author~~		
10th author	ALESSANDRA	CASTEGNA	I agree to the proposed new authorship shown in section 4 **/and the addition/~~removal*~~of my name to the authorship list** /~~and the proposed change in corresponding author~~		

Please use an additional sheet if there are more than 10 authors.

In case of author collaborations with formal agreement:

	Name of consortium/consortia	First name	Family name		Signature	Date
Representative/legal guarantor				I agree to the proposed new authorship shown in section 4 **/and the addition/removal*of my name to the authorship list** /and the proposed change in corresponding author		

Both added/removed authors should complete the information in the first table under Section 6.

---- End of form ----

SPRINGER NATURE

Change of authorship request form - Journals (pre-acceptance)

Section 6: Declaration of agreement. *All* authors, unchanged, new and removed *must* sign this declaration.
(NB: Please print the form, (docu)-sign and return/upload a scanned copy. Please note that signatures that have been inserted as an image file are acceptable as long as it is handwritten. Typed names in the signature box are unacceptable.) *** Please delete as appropriate. Delete all of the bold if you were on the original authorship list and are remaining as an author.**

	First name	Family name		Signature	Date
1st author	ANDREA	RASOLA	I agree to the proposed new authorship shown in section 4 **/and the addition/~~removal*of my~~ name to the authorship list** /~~and the proposed change in corresponding author~~	[signature]	18/03/22
2nd author			I agree to the proposed new authorship shown in section 4 **/and the addition/removal*of my name to the authorship list** /and the proposed change in corresponding author		
3rd author			I agree to the proposed new authorship shown in section 4 **/and the addition/removal*of my name to the authorship list** /and the proposed change in corresponding author		
4th authors			I agree to the proposed new authorship shown in section 4 **/and the addition/removal*of my name to the authorship list** /and the proposed change in corresponding author		
5th author			I agree to the proposed new authorship shown in section 4 **/and the addition/removal*of my name to the authorship list** /and the proposed change in corresponding author		
6th author			I agree to the proposed new authorship shown in section 4 **/and the addition/removal*of my name to the authorship list** /and the proposed change in corresponding author		
7th author			I agree to the proposed new authorship shown in section 4 **/and the addition/removal*of my name to the authorship list** /and the proposed change in corresponding author		

SPRINGER NATURE Springer Nature is one of the world's leading global research, educational and professional publishers, created in May 2015 through the combination of Nature Publishing Group, Palgrave Macmillan, Macmillan Education and Springer Science+Business Media.

SPRINGER NATURE

Change of authorship request form - Journals (pre-acceptance)

Section 6: Declaration of agreement. *All* authors, unchanged, new and removed *must* sign this declaration.
(NB: Please print the form, (docu)-sign and return/upload a scanned copy. Please note that signatures that have been inserted as an image file are acceptable as long as it is handwritten. Typed names in the signature box are unacceptable.) *** Please delete as appropriate. Delete all of the bold if you were on the original authorship list and are remaining as an author.**

	First name	Family name		Signature	Date
1st author	IONICA	MASGRAS	I agree to the proposed new authorship shown in section 4 /**and the addition/~~removal*of my~~ name to the authorship list** /~~and the proposed change in corresponding author~~	[illegible]	17/03/22
2nd author	GIUSEPPE	CANNINO	I agree to the proposed new authorship shown in section 4 /**and the addition/~~removal*of my~~ name to the authorship list** /~~and the proposed change in corresponding author~~		
3rd author	FRANCESCO	CISCATO	I agree to the proposed new authorship shown in section 4 /**and the addition/~~removal*of my~~ name to the authorship list** /~~and the proposed change in corresponding author~~	Francesco Ciscato	17/03/22
4th authors	CARLOS	SANCHEZ-MARTIN	I agree to the proposed new authorship shown in section 4 /**and the addition/~~removal*of my~~ name to the authorship list** /~~and the proposed change in corresponding author~~	[illegible]	17/03/22
5th author	FERESHTEH	BABAEI DARVISHI	I agree to the proposed new authorship shown in section 4 /**and the addition/~~removal~~*of my name to the authorship list** /~~and the proposed change in corresponding author~~	[illegible]	17.03.2022
6th author	FRANCESCA	SCANTAMBURLO	I agree to the proposed new authorship shown in section 4 /**and the addition/~~removal~~*of my name to the authorship list** /~~and the proposed change in corresponding author~~	Francesca Scantamburlo	17/03/22
7th author	MARCO	PIZZI	I agree to the proposed new authorship shown in section 4 /**and the addition/~~removal*of my~~ name to the authorship list** /~~and the proposed change in corresponding author~~		

SPRINGER NATURE Springer Nature is one of the world's leading global research, educational and professional publishers, created in May 2015 through the combination of Nature Publishing Group, Palgrave Macmillan, Macmillan Education and Springer Science+Business Media.

Change of authorship request form - Journals (pre-acceptance)

	First name	Family name		Signature	Date
8th author	ALESSIO	MENGA	I agree to the proposed new authorship shown in section 4 /**and the addition/~~removal*of my~~ name to the authorship list** /~~and the proposed change in corresponding author~~		
9th author	DOLORES	FREGONA	I agree to the proposed new authorship shown in section 4 /**and the addition/~~removal*of my~~ name to the authorship list** /~~and the proposed change in corresponding author~~		
10th author	ALESSANDRA	CASTEGNA	I agree to the proposed new authorship shown in section 4 /**and the addition/~~removal*of my~~ name to the authorship list** /~~and the proposed change in corresponding author~~	Alessandra Ca[illegible]	17/03/22

Please use an additional sheet if there are more than 10 authors.

In case of author collaborations with formal agreement:

	Name of consortium/consortia	First name	Family name		Signature	Date
Representative/legal guarantor				I agree to the proposed new authorship shown in section 4 /**and the addition/removal*of my name to the authorship list** /and the proposed change in corresponding author		

Both added/removed authors should complete the information in the first table under Section 6.

---- End of form ----

SPRINGER NATURE

Change of authorship request form - Journals (pre-acceptance)

Section 6: Declaration of agreement. *All* authors, unchanged, new and removed *must* sign this declaration.
(NB: Please print the form, (docu)-sign and return/upload a scanned copy. Please note that signatures that have been inserted as an image file are acceptable as long as it is handwritten. Typed names in the signature box are unacceptable.) *** Please delete as appropriate. Delete all of the bold if you were on the original authorship list and are remaining as an author.**

	First name	Family name		Signature	Date
1st author	IONICA	MASGRAS	I agree to the proposed new authorship shown in section 4 **/and the addition/removal*of my name to the authorship list /and the proposed change in corresponding author**	Ionica Masgras	17/03/22
2nd author	GIUSEPPE	CANNINO	I agree to the proposed new authorship shown in section 4 **/and the addition/removal*of my name to the authorship list /and the proposed change in corresponding author**	Giuseppe Cannino	17/03/22
3rd author	FRANCESCO	CISCATO	I agree to the proposed new authorship shown in section 4 **/and the addition/removal*of my name to the authorship list /and the proposed change in corresponding author**	Francesco Ciscato	17/03/22
4th authors	CARLOS	SANCHEZ-MARTIN	I agree to the proposed new authorship shown in section 4 **/and the addition/removal*of my name to the authorship list /and the proposed change in corresponding author**		
5th author	FERESHTEH	BABAEI DARVISHI	I agree to the proposed new authorship shown in section 4 **/and the addition/removal*of my name to the authorship list /and the proposed change in corresponding author**	[illegible]	17.03.2022
6th author	FRANCESCA	SCANTAMBURLO	I agree to the proposed new authorship shown in section 4 **/and the addition/removal*of my name to the authorship list /and the proposed change in corresponding author**	Francesca Scantamburlo	17/03/22
7th author	MARCO	PIZZI	I agree to the proposed new authorship shown in section 4 **/and the addition/removal*of my name to the authorship list /and the proposed change in corresponding author**		

SPRINGER NATURE Springer Nature is one of the world's leading global research, educational and professional publishers, created in May 2015 through the combination of Nature Publishing Group, Palgrave Macmillan, Macmillan Education and Springer Science+Business Media.

	First name	Family name		Signature	Date
8th author	ALESSIO	MENGA	I agree to the proposed new authorship shown in section 4 /**and the addition/~~removal*of my~~ name to the authorship list** /~~and the proposed change in corresponding author~~		
9th author	DOLORES	FREGONA	I agree to the proposed new authorship shown in section 4 /**and the addition/~~removal*of my~~ name to the authorship list** /~~and the proposed change in corresponding author~~	[signature]	17/03/22
10th author	ALESSANDRA	CASTEGNA	I agree to the proposed new authorship shown in section 4 /**and the addition/~~removal*of my~~ name to the authorship list** /~~and the proposed change in corresponding author~~		

Please use an additional sheet if there are more than 10 authors.

In case of author collaborations with formal agreement:

	Name of consortium/consortia	First name	Family name		Signature	Date
Representative/legal guarantor				I agree to the proposed new authorship shown in section 4 /**and the addition/removal*of my name to the authorship list** /and the proposed change in corresponding author		

Both added/removed authors should complete the information in the first table under Section 6.

---- End of form ----

SPRINGER NATURE

Change of authorship request form - Journals (pre-acceptance)

Section 6: Declaration of agreement. *All* authors, unchanged, new and removed *must* sign this declaration.
(NB: Please print the form, (docu)-sign and return/upload a scanned copy. Please note that signatures that have been inserted as an image file are acceptable as long as it is handwritten. Typed names in the signature box are unacceptable.) *** Please delete as appropriate. Delete all of the bold if you were on the original authorship list and are remaining as an author.**

	First name	Family name		Signature	Date
1st author	IONICA	MASGRAS	I agree to the proposed new authorship shown in section 4 **/and the addition/removal*of my name to the authorship list** /and the proposed change in corresponding author	Ionica Masgras	17/03/22
2nd author	GIUSEPPE	CANNINO	I agree to the proposed new authorship shown in section 4 **/and the addition/removal*of my name to the authorship list** /and the proposed change in corresponding author		
3rd author	FRANCESCO	CISCATO	I agree to the proposed new authorship shown in section 4 **/and the addition/removal*of my name to the authorship list** /and the proposed change in corresponding author	Francesco Ciscato	17/03/22
4th authors	CARLOS	SANCHEZ-MARTIN	I agree to the proposed new authorship shown in section 4 **/and the addition/removal*of my name to the authorship list** /and the proposed change in corresponding author		
5th author	FERESHTEH	BABAEI DARVISHI	I agree to the proposed new authorship shown in section 4 **/and the addition/removal*of my name to the authorship list** /and the proposed change in corresponding author	[illegible]	17.03.2022
6th author	FRANCESCA	SCANTAMBURLO	I agree to the proposed new authorship shown in section 4 **/and the addition/removal*of my name to the authorship list** /and the proposed change in corresponding author	Francesca Scantamburlo	17/03/22
7th author	MARCO	PIZZI	I agree to the proposed new authorship shown in section 4 **/and the addition/removal*of my name to the authorship list** /and the proposed change in corresponding author	[illegible]	18/03/22

SPRINGER NATURE Springer Nature is one of the world's leading global research, educational and professional publishers, created in May 2015 through the combination of Nature Publishing Group, Palgrave Macmillan, Macmillan Education and Springer Science+Business Media.

SPRINGER NATURE

Change of authorship request form - Journals (pre-acceptance)

Section 1: Please provide the current title of manuscript

Manuscript ID no.: CDD-21-1654

Title: TUMOR GROWTH OF NEUROFIBROMIN-DEFICIENT CELLS IS DRIVEN BY DECREASED RESPIRATION AND HAMPERED BY NAD^+ AND SIRT3

Section 2: Please provide the previous authorship, in the order shown on the manuscript before the changes were introduced. Please indicate the corresponding author by adding (CA) behind the name.

	First name(s)	Family name	ORCID or SCOPUS id, if available
1st author	IONICA	MASGRAS	
2nd author	GIUSEPPE	CANNINO	
3rd author	FRANCESCO	CISCATO	
4th author	CARLOS	SANCHEZ-MARTIN	
5th author	MARCO	PIZZI	
6th author	ALESSIO	MENGA	0000-0002-2827-5298
7th author	DOLORES	FREGONA	
8th author	ALESSANDRA	CASTEGNA	
9th author	ANDREA	RASOLA	0000-0003-4522-3008
10th author			

Please use an additional sheet if there are more than 10 authors.

SPRINGER NATURE Springer Nature is one of the world's leading global research, educational and professional publishers, created in May 2015 through the combination of Nature Publishing Group, Palgrave Macmillan, Macmillan Education and Springer Science+Business Media.

Scansionato con CamScanner

Change of authorship request form - Journals (pre-acceptance)

	First name	Family name		Signature	Date
8th author	ALESSIO	MENGA	I agree to the proposed new authorship shown in section 4 **/and the addition/~~removal*of my~~ name to the authorship list** /~~and the proposed change in corresponding author~~	[illegible]	18/3/22
9th author	DOLORES	FREGONA	I agree to the proposed new authorship shown in section 4 **/and the addition/removal~~*of my~~ name to the authorship list** /~~and the proposed change in corresponding author~~		
10th author	ALESSANDRA	CASTEGNA	I agree to the proposed new authorship shown in section 4 **/and the addition/removal~~*of my~~ name to the authorship list** /~~and the proposed change in corresponding author~~		

Please use an additional sheet if there are more than 10 authors.

In case of author collaborations with formal agreement:

	Name of consortium/consortia	First name	Family name		Signature	Date
Representative/legal guarantor				I agree to the proposed new authorship shown in section 4 **/and the addition/removal*of my name to the authorship list** /and the proposed change in corresponding author		

Both added/removed authors should complete the information in the first table under Section 6.

---- End of form ----

SPRINGER NATURE Springer Nature is one of the world's leading global research, educational and professional publishers, created in May 2015 through the combination of Nature Publishing Group, Palgrave Macmillan, Macmillan Education and Springer Science+Business Media.

Scansionato con CamScanner
